# Aluminium-based ionic liquid grafted on biochar as a heterogeneous catalyst for the selective synthesis of tetrazole and 2,3-dihydroquinazolin 4(1*H*)-one derivatives†

**DOI:** 10.1039/d3ra06440a

**Published:** 2023-12-06

**Authors:** Masoomeh Norouzi, Parisa Moradi, Maryam Khanmoradi

**Affiliations:** a Department of Chemistry, Faculty of Science, Ilam University PO Box 69315516 Ilam Iran m.norozi@ilam.ac.ir

## Abstract

2,3-Dihydroquinazolin-4(1*H*)-one and tetrazole are a class of nitrogen-containing heterocyclic compounds that play an important role in drug design and are an important part of many biological and industrial compounds. In this study, aluminium-based ionic liquid grafted onto a biochar surface (BC/[TESPMI]AlCl_4_) was synthesized and used as a catalyst for the synthesis of a series of biological derivatives including 2,3-dihydroquinazolin-4(1*H*)-ones in ethanol at reflux conditions and tetrazoles in PEG-400 at 110 °C. All products were obtained with good selectivity and yield of 90 to 97%. The catalyst was thoroughly characterized using various techniques such as thermogravimetric analysis (TGA), energy-dispersive X-ray spectroscopy (EDX), and powder X-ray diffraction (PXRD), scanning electron microscopy (SEM), inductively coupled plasm (ICP), and transmission electron microscopy (TEM), which confirmed the successful chemical grafting. This methodology has several advantages, including shorter reaction time, high yield of product, and recyclability of the catalyst. The catalyst remained active for five reaction cycles.

## Abstract 2

2,3-Dihydroquinazolin-4(1*H*)-one and tetrazole are a class of nitrogen-containing heterocyclic compounds that play an important role in drug design and are an important part of many biological and industrial compounds.

## Graphical abstract

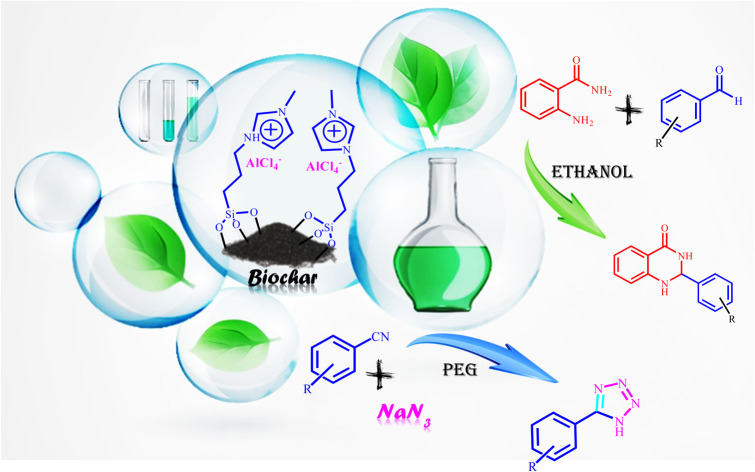

## 1. Introduction

Biomass is a valuable carbon-neutral resource with unique properties, such as tailored surface functional groups and a large surface area. It is readily available and can be used as an inexpensive starting material in various fields. One of the most important uses of biomass, is the preparation of biochar using the pyrolysis process. Biochar obtained from the pyrolysis of biomass has low porosity, high specific surface area, and limited surface functional groups.^1–3^ To overcome these problems to maximize the use of biochar, there are several ways to activation and functionalization of biochar. In the chemical activation method, fresh biochar is mixed with activation agents such as ZnCl_2_, KOH, H_2_SO_4_, K_2_CO_3_, H_3_PO_4_, *etc.*, and heated at high temperatures in an inert gas flow.^4–6^ In a physical activation method, biochar is pyrolyzed and exposed to carbon dioxide or a controlled stream-flow, or a combination of both at above 700 °C.^7^ In this way, most of the reactive points of the carbon can be removed, and the pores can open and interconnect.^8^ Therefore, its surface area can significantly be modified.^9,10^ Several factors can be affected on the chemical-activation of biochar such as the concentration and temperature of the activation agent, feedstock type, and other factors.^11^ In addition, biochar is a low-cost, big surface area, high water-holding capacity, and pyrogenic carbon-rich material.^12–19^ Therefore, these carbon-based materials have a good potential to be used as heterogeneous catalysts even in many industrial applications, and they can be used as catalysts directly.^20–23^

Besides, ionic liquids have emerged as more environmentally friendly alternative solvents than conventional solvents and have attracted major attention from researchers.^24–26^ Typically, ionic liquid formation occurred when an organic cation was combined with an inorganic or organic anion.^27^ According to qualitative and semi-qualitative studies, ionic liquids can replace conventional catalysts with the advantage that they do not cause any toxicity in the reaction mixture.^28^ Immobilization of ionic liquid on the surface of biochar has emerged as a superb platform for the synthesis of unique heterogeneous catalysts.^29^

Tetrazole compounds have been investigated for over 100 years and applied in a variety field of science such as gas-generating compositions, organic synthesis, biochemical and pharmaceutical applications, and some others.^30–32^ The low toxicity and high physiological activity of tetrazoles led to the fact that these materials continue to be highly regarded as widely used materials.^33–35^

2,3-Dihydroquinazolin-4(1*H*)-ones are a significant class of heterocyclic compounds that contain nitrogen. They have gained considerable interest due to their varied therapeutic and pharmacological properties, which include hepatoprotective, vasodilator, antidebrillatory, antipyretic antiatherosclerotic, and analgesic effects.^36^

Several reports have been published on the use of nano catalysts in the synthesis of tetrazole derivatives. For example, a review summarized the most important nano-based catalyst approaches used in the synthesis of 5-substituted 1*H*-tetrazole described by Mittal and Awasthi.^37^ Mohammad Hosein Afsarian and co-workers reported copper bis(diacetylcurcumin) 1,2-diaminobenzene Schiff base complex, SiO_2_-[Cu-BDACDABSBC] as a heterogeneous catalyst in the presence of ascorbic acid and a solution of water/i-PrOH (50 : 50, V/V) media at reflux condition is described. The yields of product have been achieved in the range of 75–95% for 3–8 hours.^38^ Dehghani and co-workers reported salen complex of Cu(ii) supported on superparamagnetic Fe_3_O_4_@SiO_2_ nanoparticles for the synthesis of tetrazole in DMF at 120 °C in 83–92% of product.^39^ Sharghi and co-workers reported 4′-phenyl-2,2′:6′,2′′-terpyridine–copper(ii) complex immobilized onto activated multi-walled carbon nanotubes [AMWCNTs-O-Cu(ii)-PhTPY] (in DMF at 70 °C, 75–95% yield of products).^40^ And the other work has been published using ZnO, Zn hydroxyapatite, Zn/Al HT (in DMF, at 120–130 °C, yields: 62–91%), FeCl_3_–SiO_2_, Sb_2_O_3_, γ-Fe_2_O_3_, BaWO_4_ Cu_2_O, CdCl_2_ and ZnS (solvent: DMF/MeOH, 2.5 mol% catalyst, 12 h).^41–45^ Many of these works have disadvantages such as low product efficiency, high reaction temperature, use of toxic solvents to promote the reaction, use of high amounts of catalyst, use of catalysts that cannot be recycled, *etc.* Also, there are several reports on the use of nano catalysts for the synthesis of 2,3-dihydroquinazolin-4(1*H*)-one derivatives such as solvent-free, mechanochemically scalable synthesis of 2,3-dihydroquinazolin-4(1*H*)-one using Brønsted acid catalyst reported by Gauravi Yashwantrao and co-workers (homogenous catalyst).^46^ The other strategies for the preparation of these compounds have been reported by Sun *et al.* in 2018 (110 °C, DMSO, 12 h)^47^ and Pathare *et al.* in 2019 (toluene, R.T, 2 h).^48^ Aqueous Facile Synthesis of 2,3-Dihydroquinazolin-4(1*H*)-One Derivatives by Reverse Zinc Oxide was reported by Jie Mou and the other work in 2020 (homogenous catalyst, 90 °C, 5 h).^49^ However, most of these protocols have limitations, such as excess oxidant, complicated reactions, harsh reaction conditions (up to 100 °C), tedious work-ups, non-renewable and toxic solvents, non-reusable catalyst, low yields and long reaction times. All of these approaches reveal that the catalytic formation of these compounds is still challenging and that the area demands to be developed further. Consequently, based on our research on heterogeneous supported catalysts^50–53^ to introduce a catalyst that does not have any of the drawbacks stated for the mentioned catalytic systems, herein, we reported aluminium-based 1-(triethoxysilyl) propyl-3-methylimidazolium chloride ionic liquid grafted on ([TESPMI]Cl)-biochar (BC/[TESPMI]AlCl_4_) as a high active, low-cost, reusable, environmentally friendly, and a heterogeneous nature catalyst. Furthermore, the catalytic performance of BC/[TESPMI]AlCl_4_ was investigated in the synthesis of 5-substituted 1*H*-tetrazole and 2,3-dihydroquinazolin-4(1*H*)-one derivatives.

## 2. Experimental

### 2.1. Materials

1-methylimidazole, (3-chloropropyl) triethoxysilane, AlCl_3_, and all other reagents, chemicals, and solvents were purchased from Merck, Aldrich, Fluka and were used without additional purification.

### 2.2. Instrumental measurements

Powder X-ray diffraction (PXRD) of catalyst were recorded on a Holland Philips diffractometer (Cu Ka, radiation at 40 kV and 30 mA). SEM, EDS and EDS-mapping analysis were performed by using a FESEM-TESCAN MIRA3 scanning electron microscope. The thermogravimetric analysis (TGA) curves are recorded between 30 and 800 °C in air with heating rate of 10 °C min^−1^. FT-IR spectra were recorded with KBr pellets using a Bruker FT-IR spectrometer (model VRTEX 70). And the transmission electron microscopy (TEM) images were obtained by a Zeiss – EM10C microscope with operating voltage at 100 kV. The elemental Al content catalyst was determined by PerkinElmer Optima 7300D inductively coupled plasma (ICP). The BET test was performed by using a BELSORP MINI II instrument.

### 2.3. Synthesis of catalyst

Biochar was obtained by placing dried chicken manure (500 g) in a porcelain crucible and pyrolyzed at the common temperature range of pyrolysis means 400 °C to 800 °C. We reached the required temperature after approximately 30 minutes of heating with the carrier N_2_ gas that scanned at 0.3 L min^−1^. In continuous, it introduced the porcelain crucibles into the heating zone with a 0.03 L min^−1^ rate of nitrogen flow. This process continues for 1 and then 2 hours. After that, the heating zone of porcelain crucibles was cut down, and cooled with N_2_ sweep with 0.3 L min^−1^ for 30 minutes. Resulting, a black solid is obtained which is biochar.^54^

In a round-bottomed flask equipped with a condenser, a mixture of 1-methylimidazole (1 mmol) and (3-chloropropyl) triethoxysilane (1 mmol) was reflexed at 100 °C for 12 h, according to recent research.^26^ After that, the reaction mixture was washed with *n*-hexane several times to get a pure yellowish viscous ionic liquid ([TESPMI]Cl).

BC/[TESPMI]Cl was prepared by dissolving 1.5 mmol of [TESPMI]Cl and 1 g of biochar in toluene and stirred at 90 °C for 24 h. The resulting product was purified by washing with EtOH for several times and drying at 60 °C and labeled as BC/[TESPMI]Cl.

The ionic liquid content (weight stability) was determined by gravimetric method after complete drying. The amount of [TESPMI]Cl per gram of support was measured to be 1.13 mmol g^−1^.

Finally, the nanocatalyst was prepared by dissolving of 1 mmol of AlCl_3_ and 0.5 g of BC/[TESPMI]Cl in EtOH (20 mL), and the reaction mixture was stirred at 60 °C for 20 h. The desirable product was obtained after filtering, washing with the mixture of ethanol/water, and dried at room temperature. This product labeled as BC/[TESPMI]AlCl_4_.

### 2.4. General procedures for preparation of 5-substituted-1*H*-tetrazoles

5-Substituted-1*H*-tetrazoles were synthesized from a mixture of 1 mmol of benzonitrile and 1.2 mmol of sodium azide in PEG-400 solvent in the presence of 50 mg BC/[TESPMI]AlCl_4_ for enough time at 110 °C. The end of reactions has been determined by TLC method and the obtained data for several derivatives were summarized in Table 2. In the end of each reaction, hot ethyl acetate has been added to the reaction mixture, and BC/[TESPMI]AlCl_4_ catalyst has been separated from the reaction mixture by a simple filtration. After that, the reaction mixture was transferred to a decanter funnel containing water, HCl and ethyl acetate. The combined organic layers were dried over anhydrous Na_2_SO_4_, then filtered, and the solvent was removed under reduced pressure and finally the crude product was purified using a silica gel plate.

### 2.5. General procedures for preparation of 2,3-dihydroquinazolin-4(1*H*)-ones

To a mixture of 2-aminobenzamide (1 mmol) and aldehyde (1 mmol) in ethanol (5 mL), 30 mg of BC/[TESPMI]AlCl_4_ was added and stirred under reflux condition for appropriate reaction time (Table 4). The end of the reaction was determined by TLC. After this, the reaction mixture was cooled down and dissolved in hot ethanol. Then it filtered off and dried at 50 °C. The product was further purified by re-crystallization form water/ethanol mixture (1 : 1).

## 3. Results and discussion

**Scheme 1:**
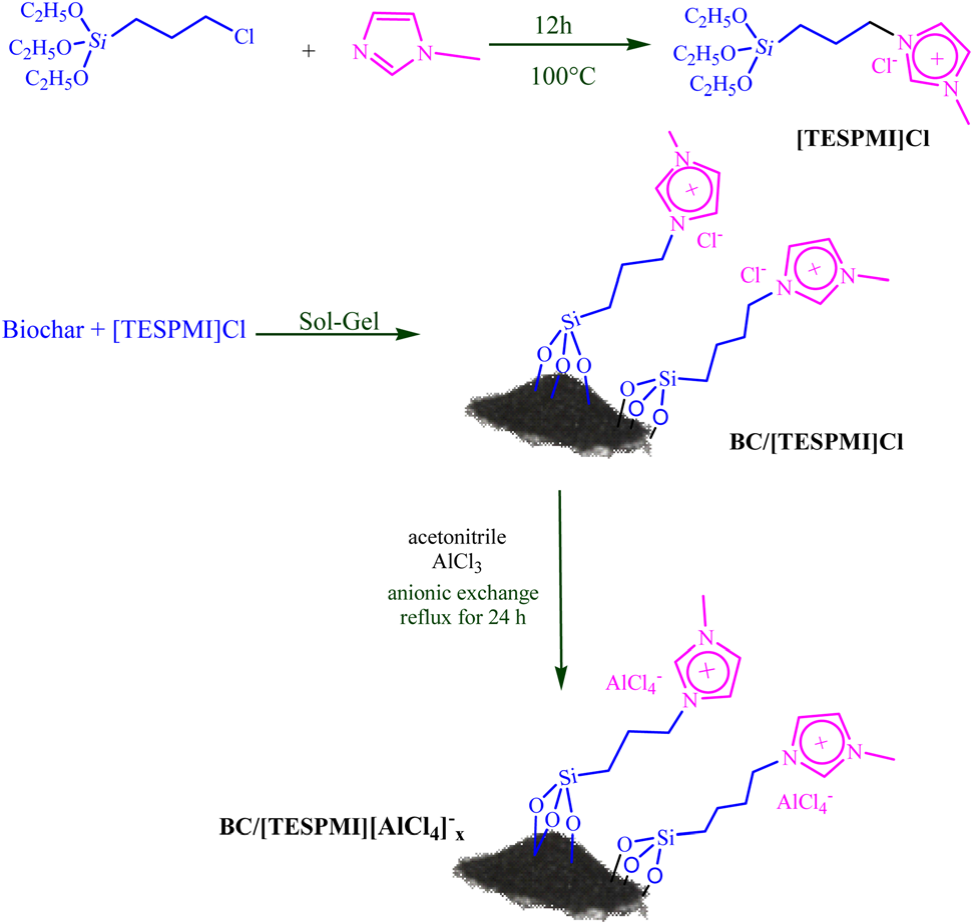
General scheme for preparation of BC/[TESPMI]AlCl_4_ catalyst.

The BC/[TESPMI]AlCl_4_ nanocatalyst was afforded according to a three-step operationally simple process, as shown in Scheme 1. It involves the discrete synthesis of a biochar and ionic liquid ([TESPMI]Cl) followed by its immobilization on the prepared support (BC/[TESPMI]Cl) and further complexation with the metal salt to obtain the final immobilized aluminum (BC/[TESPMI]AlCl_4_).^72,73^ The synthesized BC/[TESPMI]AlCl_4_ catalyst has been characterized by various techniques such as BET, SEM, FT-IR, EDAX, TGA, WDX, XRD, ICP and TEM.

### 3.1. Catalyst characterizations

**Fig. 1:**
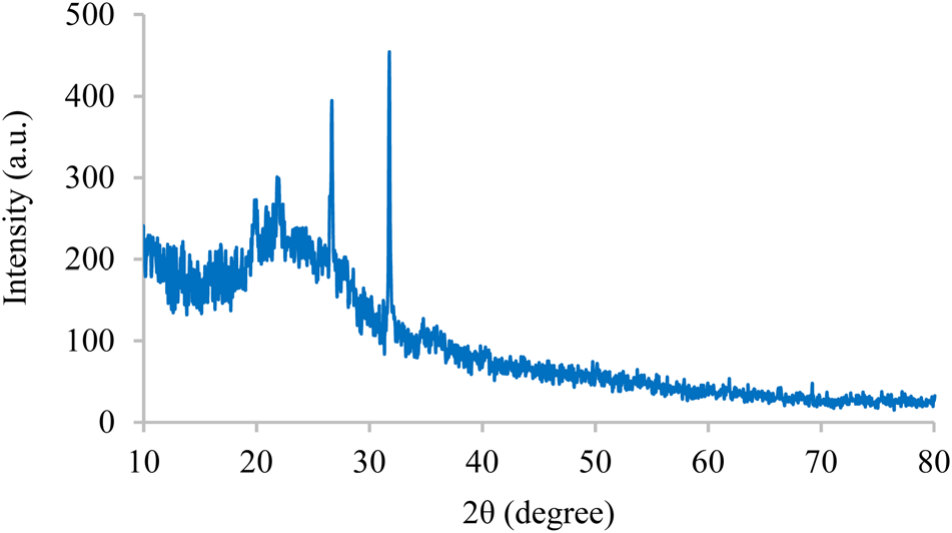
The XRD pattern of BC/[TESPMI]AlCl_4_ catalyst.

The internal structures of BC/[TESPMI]AlCl_4_ was examined by XRD analyses, as shown in Fig. 1. The XRD pattern of biochar shows broad diffraction peaks around 2*θ* = 17–30°, which reflects amorphous carbon.^54,55^ Furthermore, peaks observed at 2*θ* = 19.87°, 21.90°, 26.66° and 31.74° in the XRD pattern of BC/[TESPMI]AlCl_4_ confirm the synthesis of the catalyst.

**Fig. 2:**
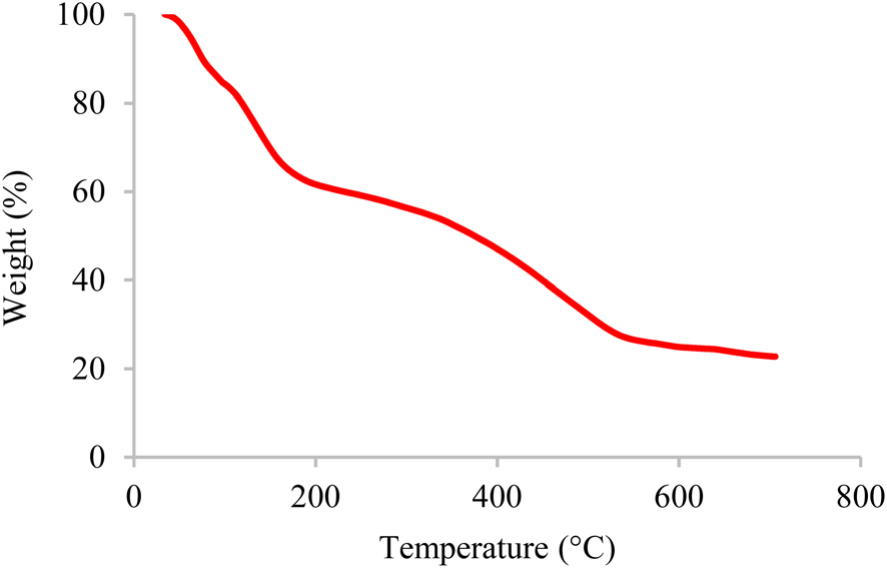
The TGA curve of BC/[TESPMI]AlCl_4_ catalyst.

The thermal stability of inorganic and organic components can be obtained by thermogravimetric analysis (TGA) at different temperatures. Therefore, this technique was used to identify the thermal stability of the BC/[TESPMI]AlCl_4_ catalyst, which results are shown in Fig. 2. As shown in Fig. 2, three stages of weight loss can be seen in the TGA diagram of catalyst, which correspond to 18%, 20%, and 27% of the weight, respectively. The first mass loss of catalyst is corresponding to the release of water and other solvents from the nanocatalyst network.^54^ The second weight loss is related to the elimination of organic groups that are immobilized on the surface of biochar.^54^ The third mass loss are corresponding to the continued biochar pyrolysis.

**Fig. 3:**
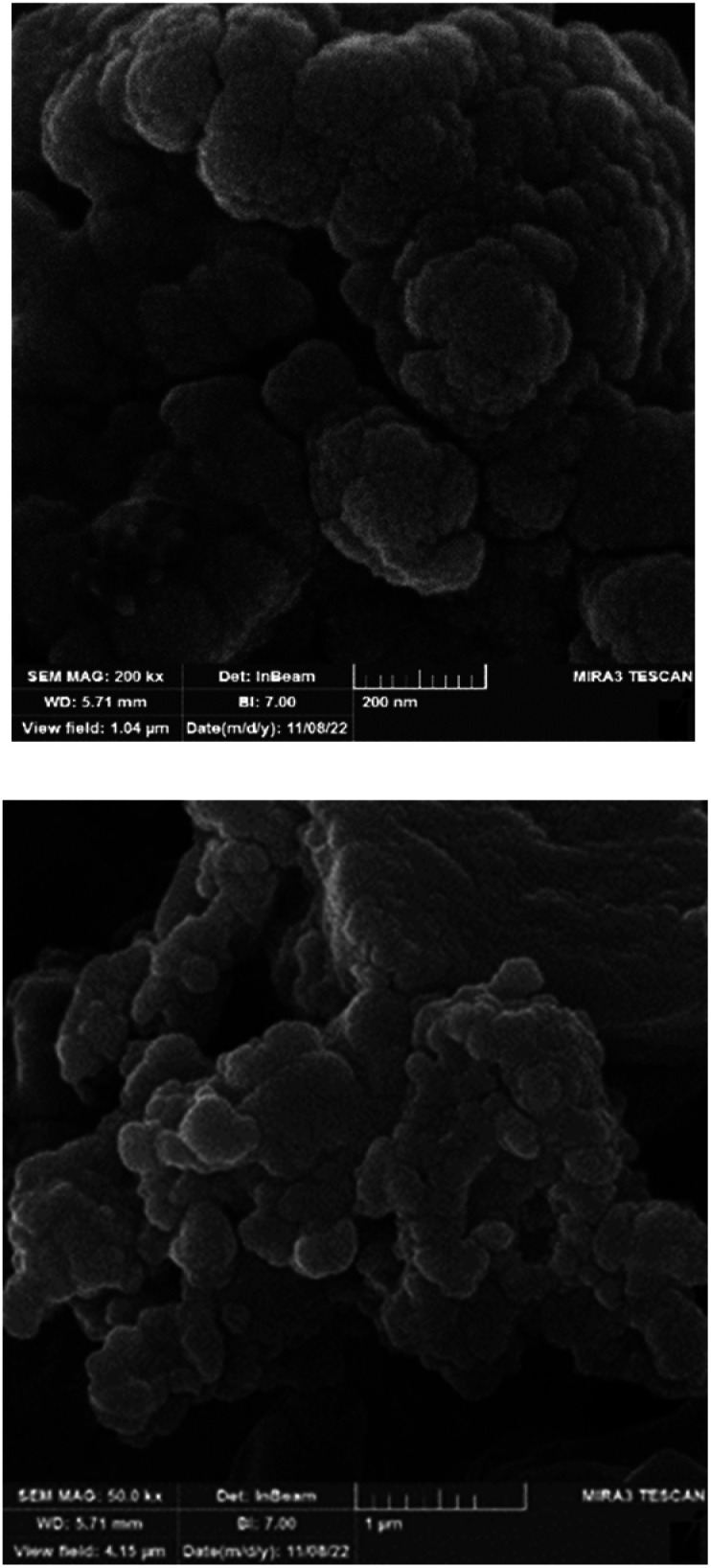
The SEM images of BC/[TESPMI]AlCl_4_ catalyst.

The morphology of BC/[TESPMI]AlCl_4_ catalyst was explored using scanning electron microscopy (SEM) technique and depicted in Fig. 3. As shown in Fig. 3, the dense and interconnected nanoparticles with a nanometer size have a quasi-spherical morphology uniformly. These results confirm that biochar has not undergone any changes during the modification.

**Fig. 4:**
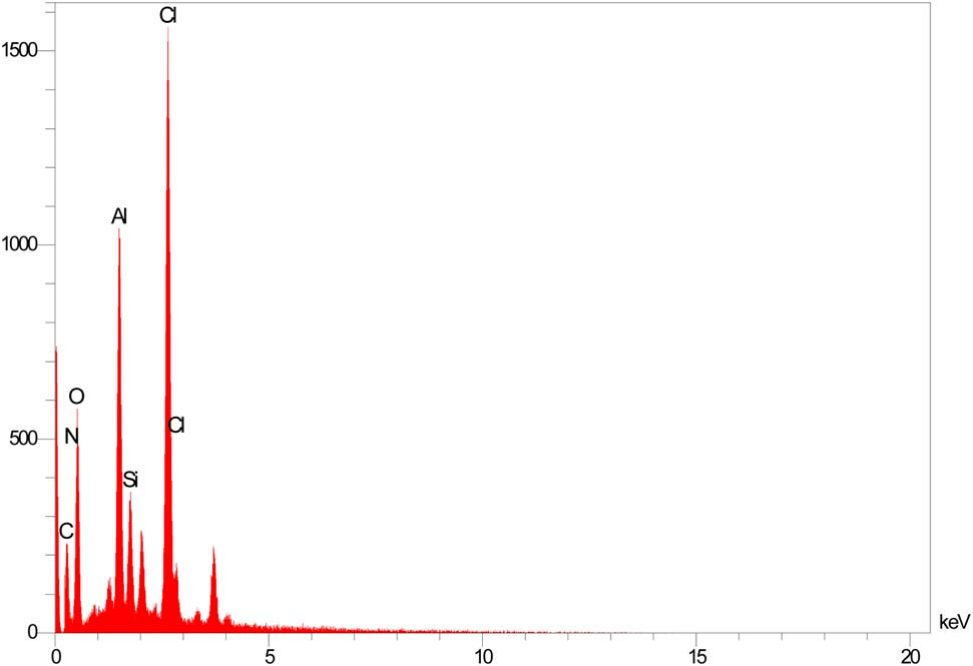
EDS spectrum of BC/[TESPMI]AlCl_4_ catalyst.

Energy-dispersive X-ray spectroscopy (EDS) is widely used in the characterizations of the content of different elements in the nanostructure's frameworks. Based on this analysis, C, Cl, Si, O, N and Al species are present on the structure of the catalyst (Fig. 4).

**Fig. 5:**
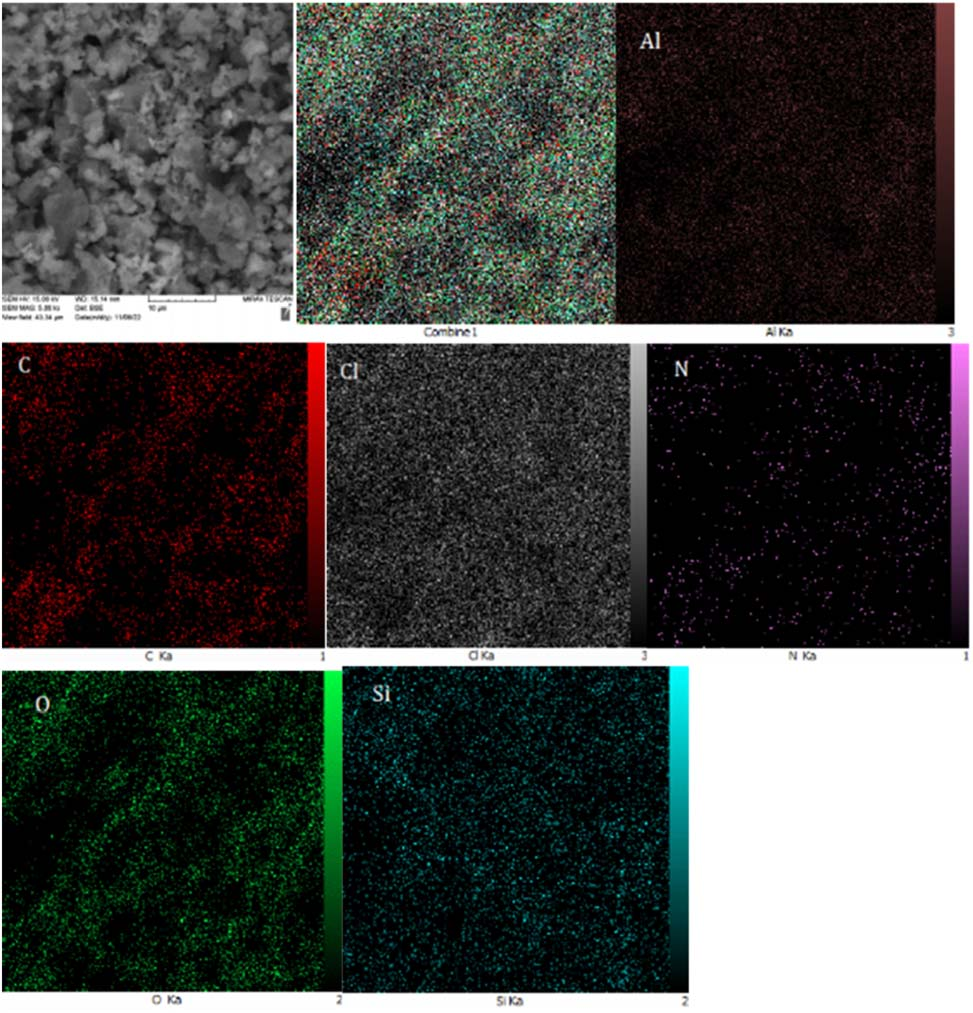
The WDX analysis for the BC/[TESPMI]AlCl_4_ catalyst.

To further characterize the catalyst, wavelength dispersive X-ray spectroscopy (WDX) was employed to determine the elemental distributed of BC/[TESPMI]AlCl_4_ catalyst, as illustrated in Fig. 5. This analysis showed that all elements were homogeneously distributed in the structure of BC/[TESPMI]AlCl_4._

The exact amount of aluminium in BC/[TESPMI]AlCl_4_ was measured by inductively coupled plasma optical emission spectroscopy (ICP-OES) analysis. Based on the ICP results, 0.73 mmol of Al was loaded onto 1.0 g of the catalyst which shows a high loading capacity for this nanocatalyst.

**Fig. 6:**
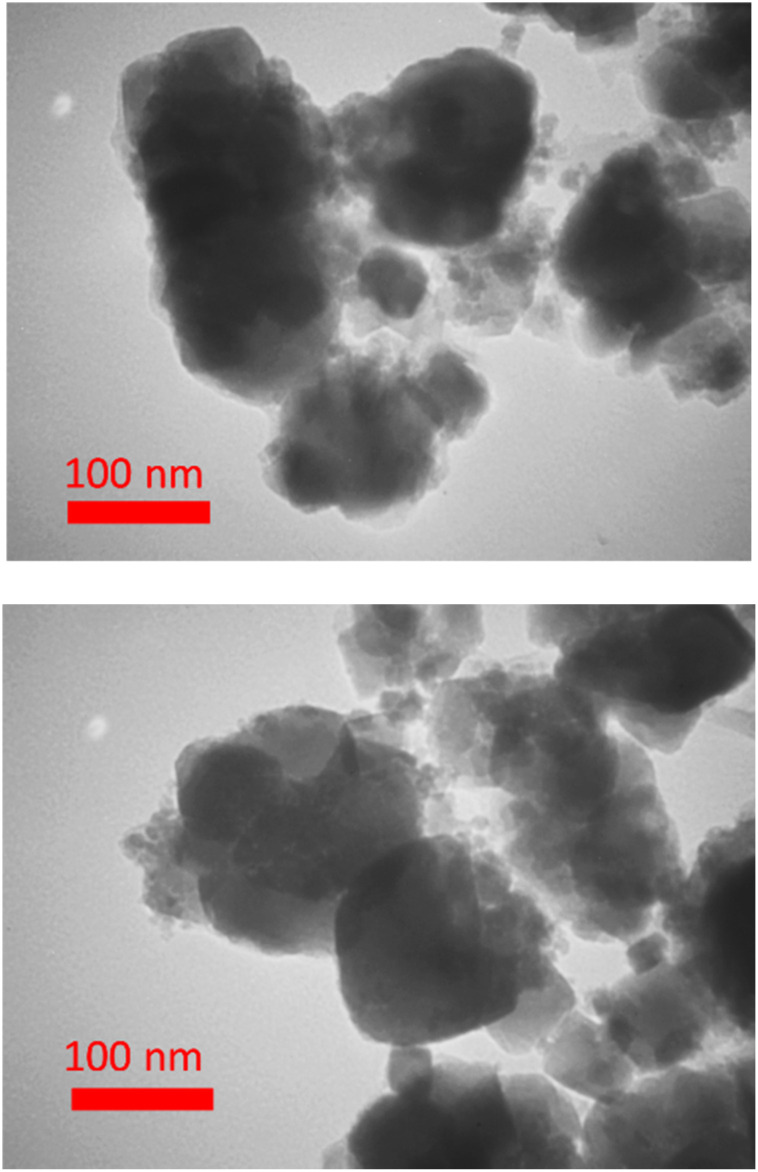
Representative TEM micrographs of BC/[TESPMI]AlCl_4_ catalyst.

Also, the microscopic analysis of BC/[TESPMI]AlCl_4_ was investigated by TEM (Fig. 6). The TEM technique showed that most biochar particles were roughly spherical, and its size distribution diagram shows a uniform size distribution with an average size less than 100 nm.

**Fig. 7:**
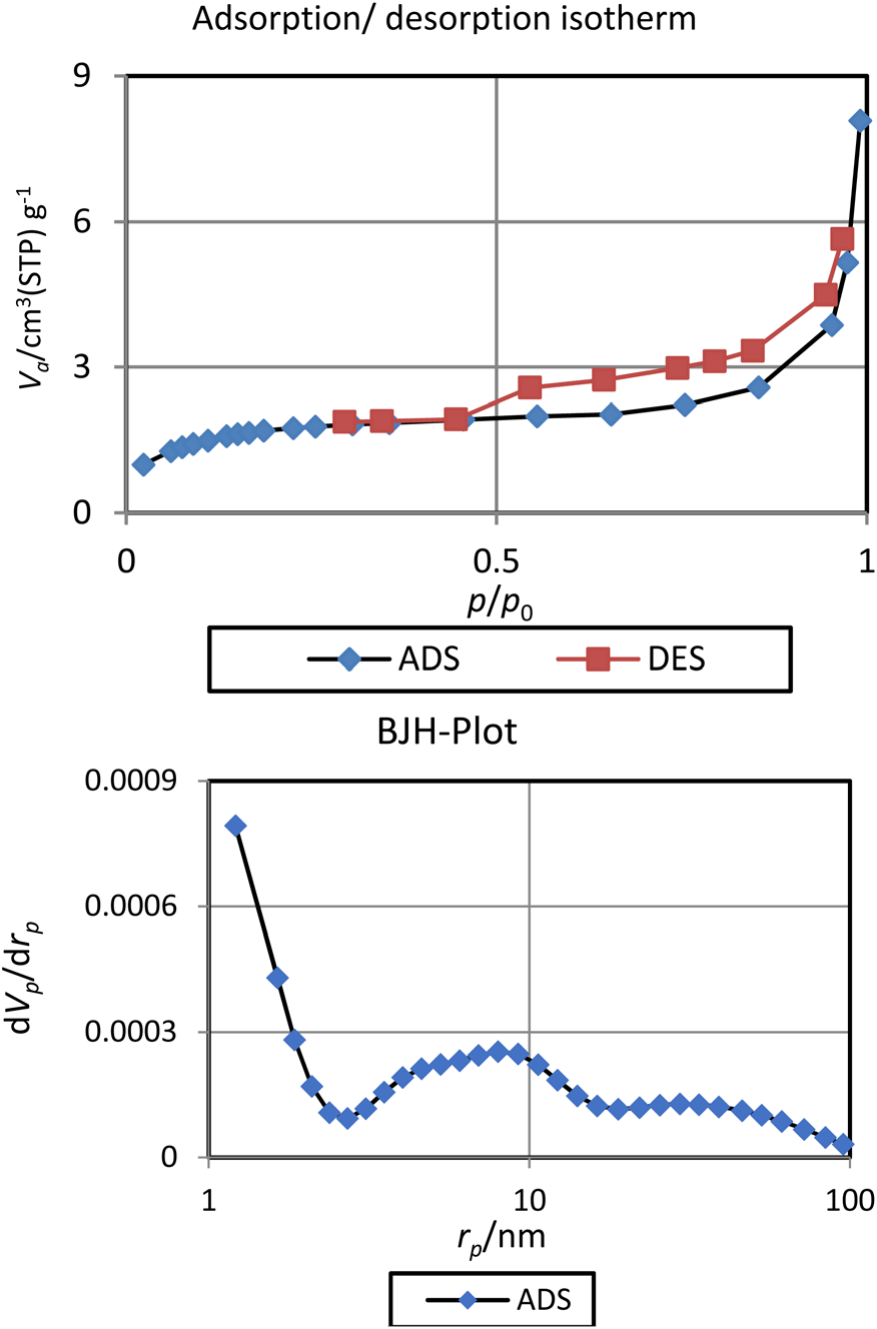
N_2_ adsorption–desorption isotherms and BJH-Plot of BC/[TESPMI]AlCl_4_ catalyst.

The porosity properties of BC/[TESPMI]AlCl_4_ catalyst were explored by the nitrogen ads/des isotherm analysis (Fig. 7). According to previous reports on these analyses of biochar materials, the surface area and pore volume of BC/[TESPMI]AlCl_4_ catalyst are lower than surface area and pore volume of biochar. The values of these parameters for this catalyst are 6 m^2^ g^−1^ (surface area) and 0.01 cm^3^ g^−1^ (total pore volume). Meanwhile, the pore diameter value of BC/[TESPMI]AlCl_4_ catalyst is 8.0 nm, which is higher than of the BC substrate.^54^ Considering that the pore diameter of BC/[TESPMI]AlCl_4_ catalyst is between 2 and 50 nm.

**Fig. 8:**
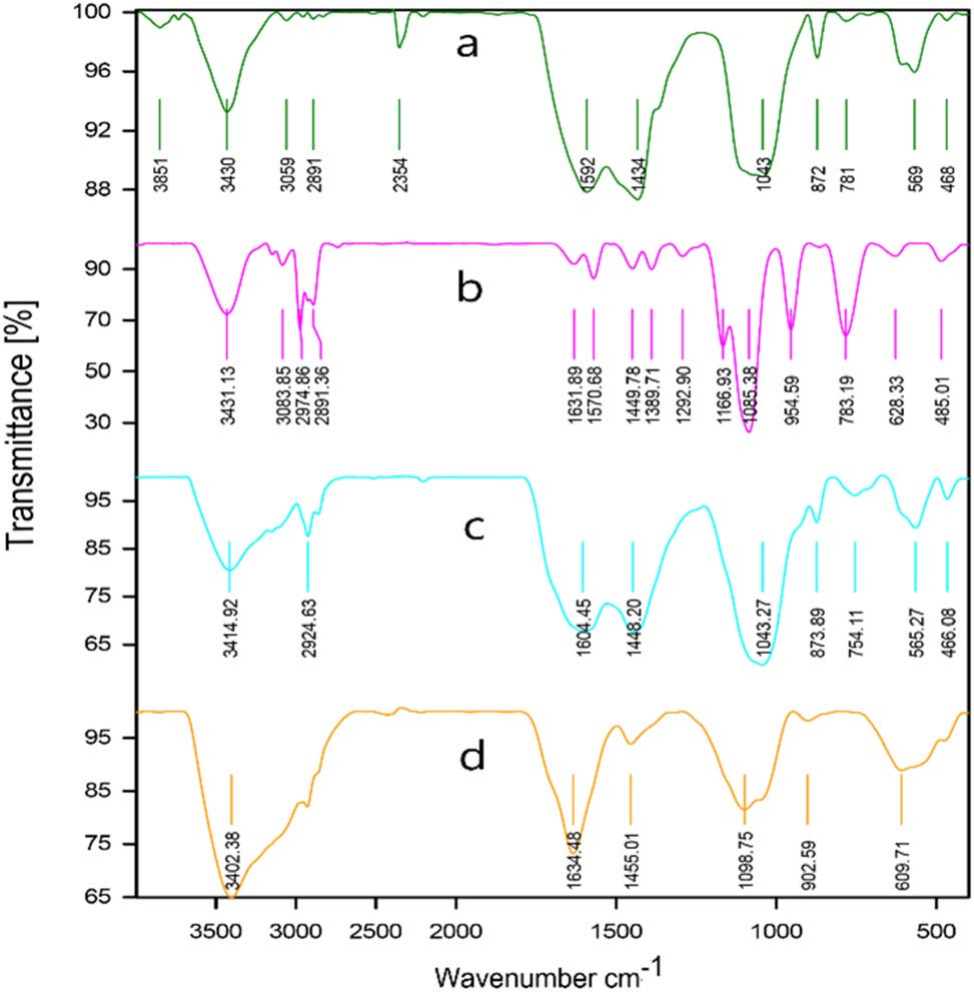
The FT-IR spectra for (a) biochar (BC), (b) [TESPMI]Cl, (c) BC/[TESPMI]Cl, and (d) BC/[TESPMI]AlCl_4_ catalyst.

To identify the functional groups in biochar (BC), [TESPMI]Cl, ionic liquid functionalized biochar (BC/[TESPMI]Cl) and BC/[TESPMI]AlCl_4_ catalyst, the FTIR spectra were taken, as shown in Fig. 8. In Fig. 8a, which corresponds to BC, several characteristic peaks can be observed at the 3430 cm^−1^ wave number, reveling oxygen-containing functional groups stretching vibrations such as phenol, alcohol, and carboxylic acid groups. The peaks around 3000 cm^−1^ are assigned to CH_2_ stretching vibrations.^54^ The IR spectrum of [TESPMI]Cl (Fig. 8b) shows absorption bands at 3431, 3083, 2974 and 2891 cm^−1^, which are related to the asymmetrical stretching of amine salt, CH_2_ units in the imidazolium ring and aliphatic groups, respectively. The peaks at 1631 and 1570 cm^−1^ are associated with the stretching vibrations of C=N and C=C groups in imidazolium rings, respectively. In addition, the bands at the interval 1085, 783 and 485 cm^−1^ are attributed to the stretching vibrations of Si–O–Si. As can be observed in Fig. 8c, the peaks at 1602 cm^−1^ and 3405 cm^−1^ are related to the presence of –C=N– and O–H groups on the surface of BC/[TESPMI]Cl. In Fig. 8d, some of the peaks that existed in the previous stage (Fig. 8c) have shifted to other frequencies and appeared a new peak at 609 cm^−1^ and 904 cm^−1^ which confirms the formation of BC/[TESPMI]AlCl_4_ catalyst.

### 3.2. Catalytic study

The catalytic performance of BC/[TESPMI]AlCl_4_ catalyst was investigated in the synthesis of 1*H*-tetrazole derivatives. First, optimization of the reaction condition is considered in the reaction between benzonitrile and sodium azide. In this stage, the effect of solvent, amount of catalyst, and temperature were examined to the synthesis of corresponding tetrazole.

First, the amount of the catalyst was optimized as an important factor in catalytic reactions. In the presence of 20 mg of the catalyst, the reaction afforded a 67% yield (Table 1, entry 1). Therefore, higher amounts of the catalyst were checked (Table 1, entries 2–4). Among different amounts of the catalyst (30, 40, and 50 mg), it was found that the best results were observed in the presence of 50 mg of BC/[TESPMI]AlCl_4_ catalyst. After optimizing the amount of catalyst, we attempted to optimize the best solvent to perform this reaction in the presence of 50 mg of BC/[TESPMI]AlCl_4_ (Table 1, entries 4–7). Our findings revealed that the solubility of sodium azide plays a significant role in the yield of the desired product. In aprotic polar and nonpolar solvents, the reaction resulted in a very low yield, likely due to the poor solubility of sodium azide. However, in polar protic solvents, we observed a higher yield of the corresponding triazole (Table 1, entries 6 and 7). Among the solvents examined, PEG-400 was found to be the best reaction medium (Table 1, entry 4).

**Table 1:** Definition of conditions for the cycloaddition reaction of sodium azide (1.2 mmol) and benzonitrile (1 mmol) in the presence of BC/[TESPMI]AlCl_4_ nanocatalyst

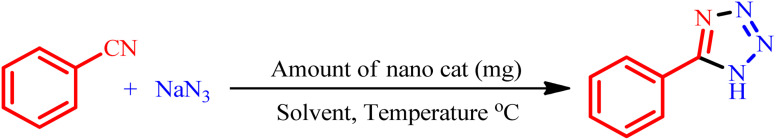					
Entry	Amount of the catalyst (mg)	Solvent	Temperature (°C)	Time (min)	Yield^a^ (%)
1	20	PEG-400	110	230	67
2	30	PEG-400	110	230	80
3	40	PEG-400	110	150	89
4	50	PEG-400	110	100	96
5	50	EtOH	Reflux	100	Trace
6	50	H_2_O	Reflux	100	40
7	50	Glysrole : coline chloride (1 : 1)	110	100	45
8	50	PEG-400	80	100	52

a Isolated yield.

**Table 2:** Preparation of 1*H*-tetrazole derivatives using BC/[TESPMI]AlCl_4_

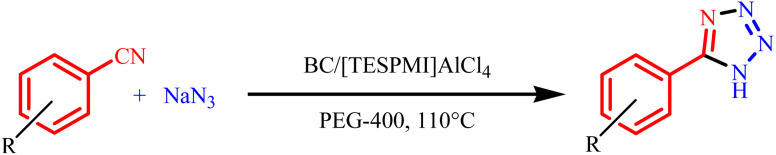				
Entry	Nitrile	Product	Time (min)	Yield^a^ (%)
1	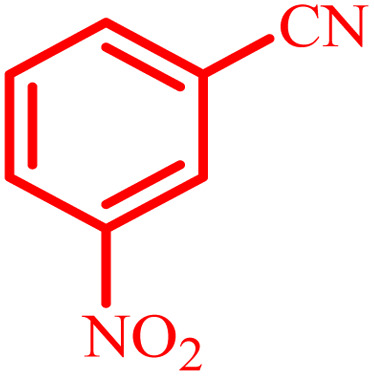	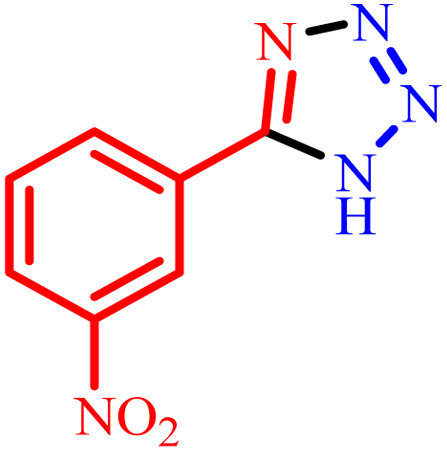	480	90
2	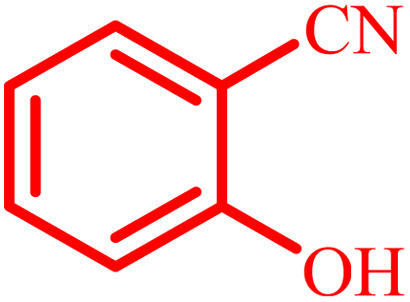	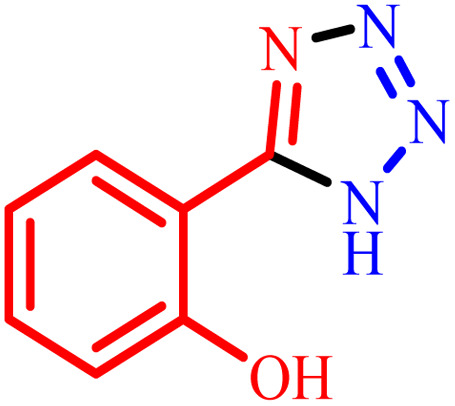	20	97
3	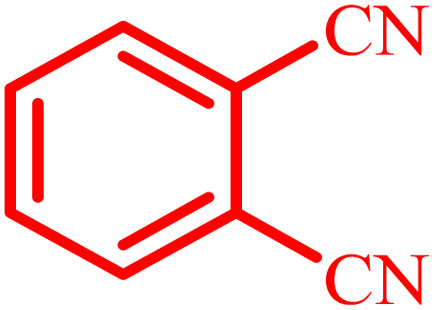	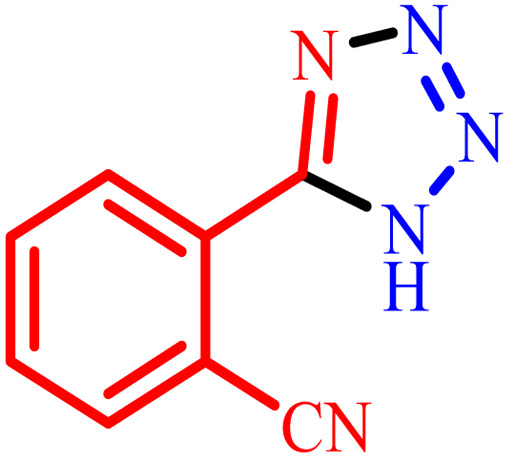	60	94
4	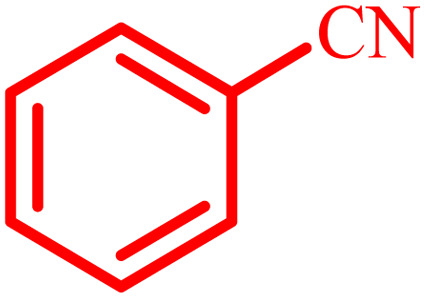	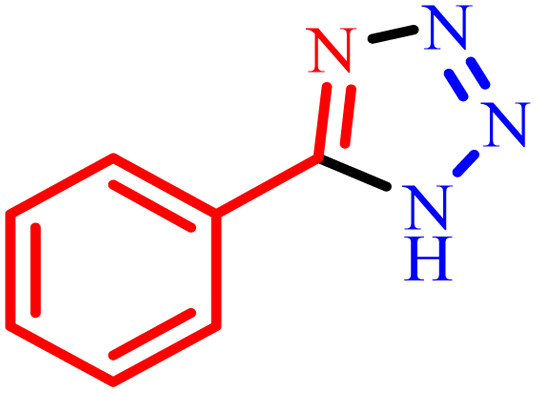	100	96
5	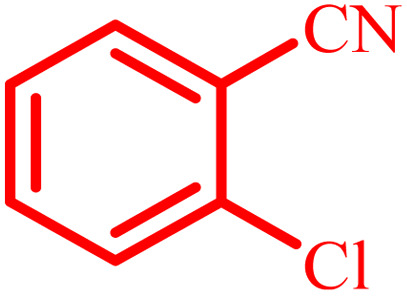	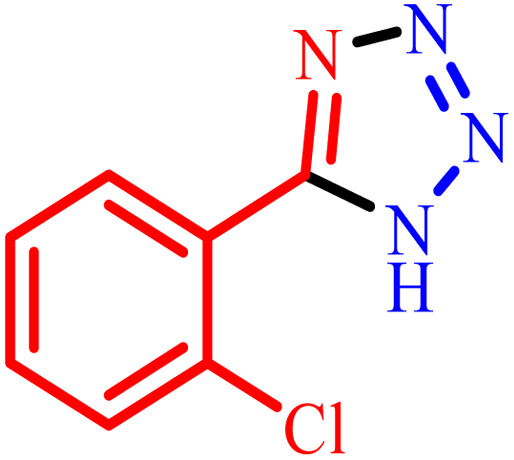	60	95
6	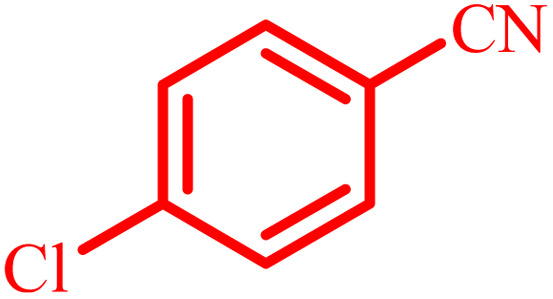	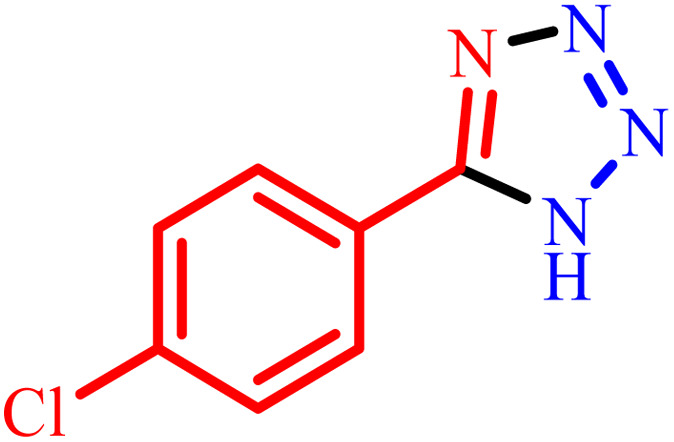	360	92
7	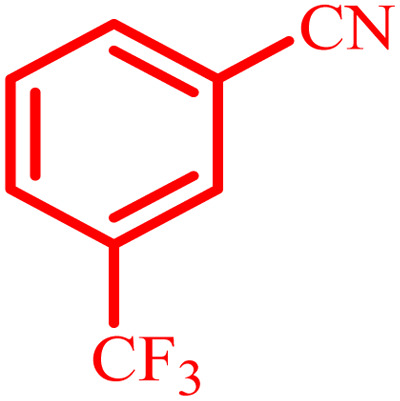	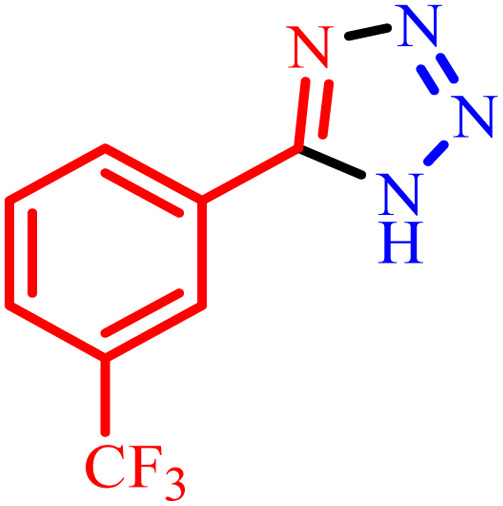	100	95

a Isolated yield.

Further the reaction temperature was optimized. As shown in Table 1 (entries 4 and 8), at low temperatures, the reaction rate was found to be very slow. Finally, 110 °C provided the best results in the synthesis of tetrazole products. Therefore, the best conditions for this selected reaction model in the presence of BC/[TESPMI]AlCl_4_ catalyst were obtained using 50 mg of the catalyst at 110 °C in PEG-400 as solvent.

Further, several benzonitrile derivatives were reacted with sodium azide in the presence of BC/[TESPMI]AlCl_4_ catalyst under optimized conditions. The benzonitrile derivatives with different electron-donating or electron-withdrawing functional groups were investigated and produced corresponding tetrazoles with excellent yields (Table 2).

Also, the catalytic efficiency of BC/[TESPMI]AlCl_4_ catalyst has been studied for the synthesis of 2,3-dihydroquinazolin-4(1*H*)-one derivatives. To get the optimized reaction conditions, several parameters have been explored in the reaction condensation of 4-chlorobenzaldehyde and 2-aminobenzamide.

To investigate the role of the solvent in promoting the reaction and find the most suitable amount of the catalyst, the reaction was carried out in the absent of BC/[TESPMI]AlCl_4_ catalyst and in the presence of biochar as catalyst. The results show that there was no reaction in either of these two reactions even after long reaction time (Table 3, entries 1 and 2). Next, we investigated the role of BC/[TESPMI]AlCl_4_ as catalyst by performing different amounts of this catalyst including 45, 30, 15, and 5 mg (Table 3, entries 3–6). The results show that 30 mg of BC/[TESPMI]AlCl_4_ revealed 92% yield of desired product. Significant efficiency was not obtained in the presence of lower amounts of the catalyst.

**Table 3:** Evaluation of the reaction parameter on the synthesis of 2,3-dihydroquinazolin-4(1*H*)-one derivatives over the catalysis of (BC/[TESPMI]AlCl_4_)

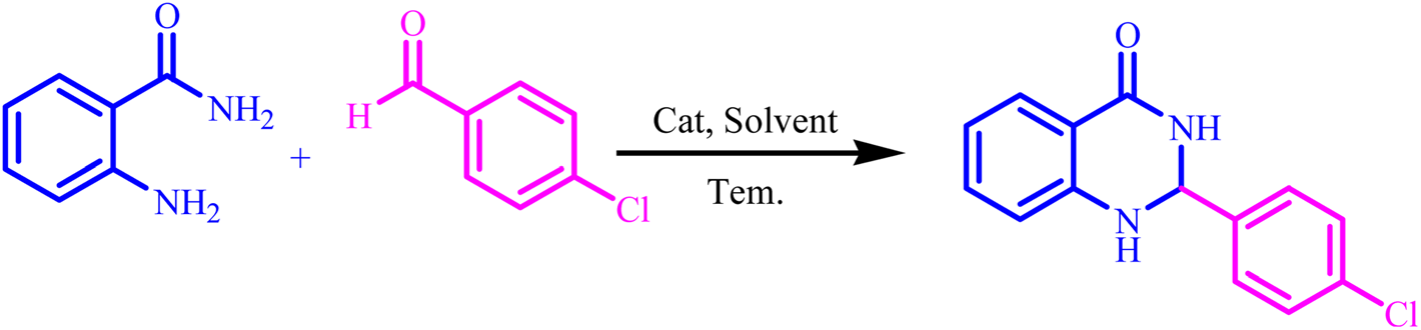						
Entry	Catalyst	Catalyst (mg)	Solvent	Temperature (°C)	Time (min)	Yield^a^^,^^b^ (%)
1	—	—	EtOH	Reflux	5 h	N.R
2	Biochar	45	EtOH	Reflux	120	Trace
3	BC/[TESPMI]AlCl_4_	45	EtOH	Reflux	25	93
4	BC/[TESPMI]AlCl_4_	30	EtOH	Reflux	25	92
5	BC/[TESPMI]AlCl_4_	15	EtOH	Reflux	60	68
6	BC/[TESPMI]AlCl_4_	5	EtOH	Reflux	90	20
7	BC/[TESPMI]AlCl_4_	30	CHCl_3_	Reflux	85	0
8	BC/[TESPMI]AlCl_4_	30	DMSO	Reflux	60	30
9	BC/[TESPMI]AlCl_4_	30	MeOH	Reflux	60	16
10	BC/[TESPMI]AlCl_4_	30	DMF	120	60	40
11	BC/[TESPMI]AlCl_4_	30	Acetonitrile	Reflux	60	35
12	BC/[TESPMI]AlCl_4_	30	DI water	Reflux	60	50
13	BC/[TESPMI]AlCl_4_	30	EtOH	50	180	15
14	BC/[TESPMI]AlCl_4_	30	EtOH	r.t	180	Trace

a Isolated yield.

b Conditions: 4-chlorobenzaldehyde (1 mmol) and 2-aminobenzamide (1 mmol), catalyst (30 mg) in EtOH solvent (5 mL).

**Table 4:** Synthesis of 2,3-dihydroquinazolin-4(1*H*)-one derivatives^a^

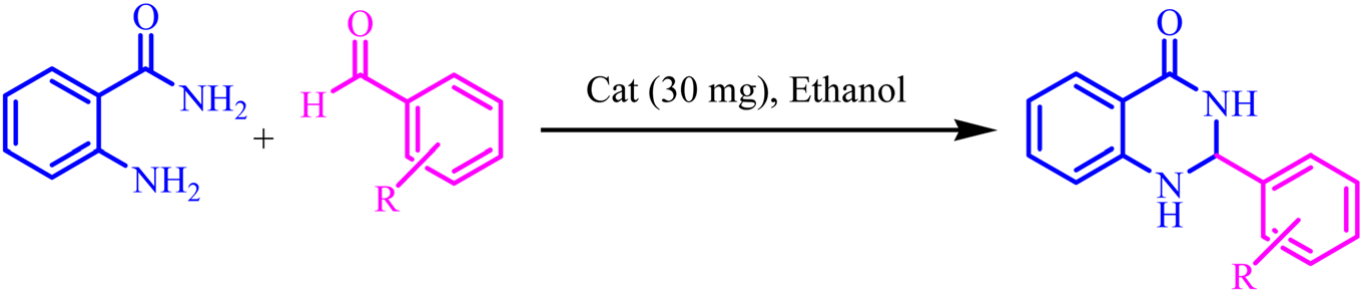				
Entry	Aldehyde	Product	Time (min)	Yield^a^ (%)
1	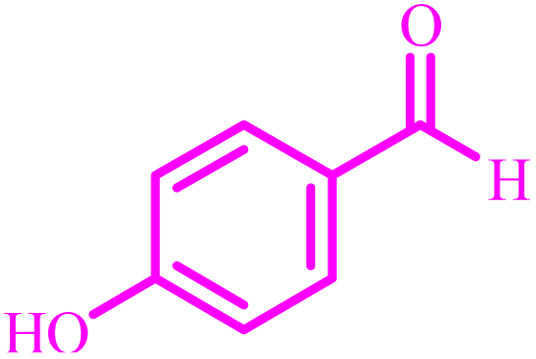	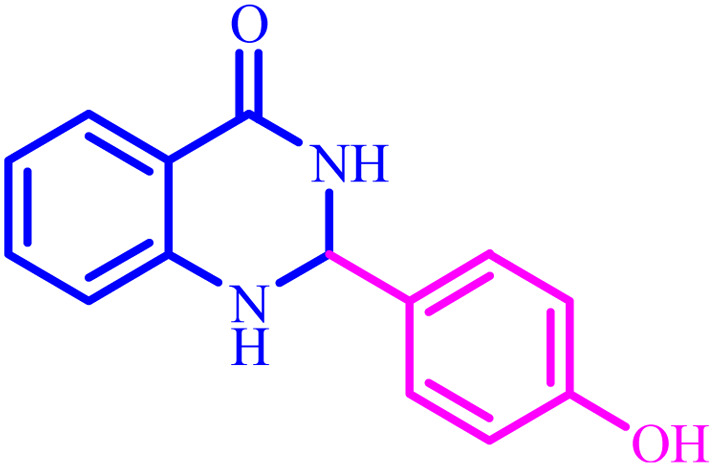	45	92
2	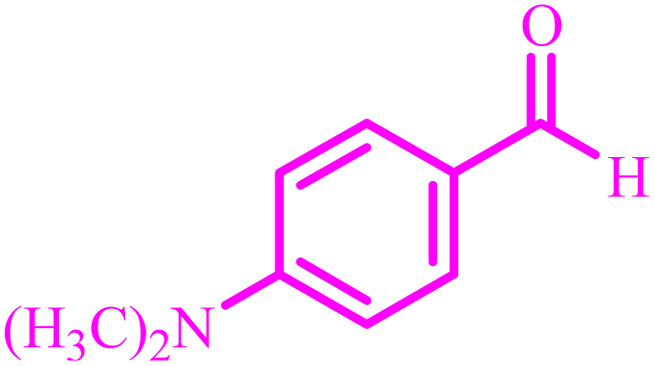	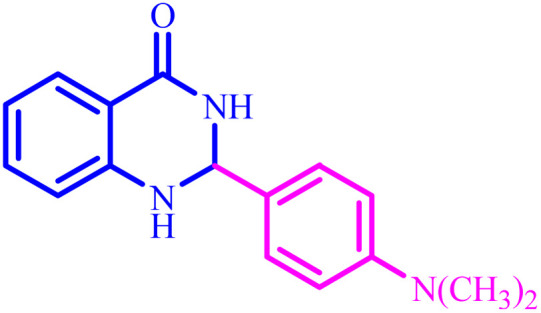	70	94
3	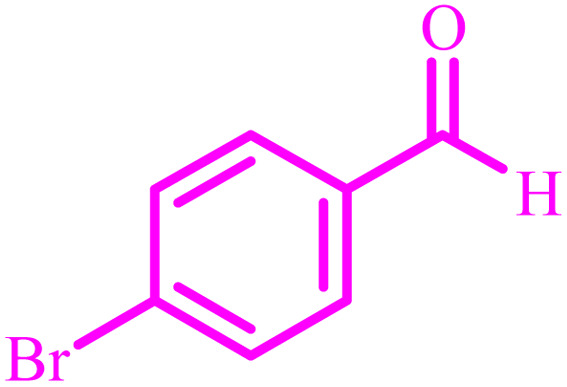	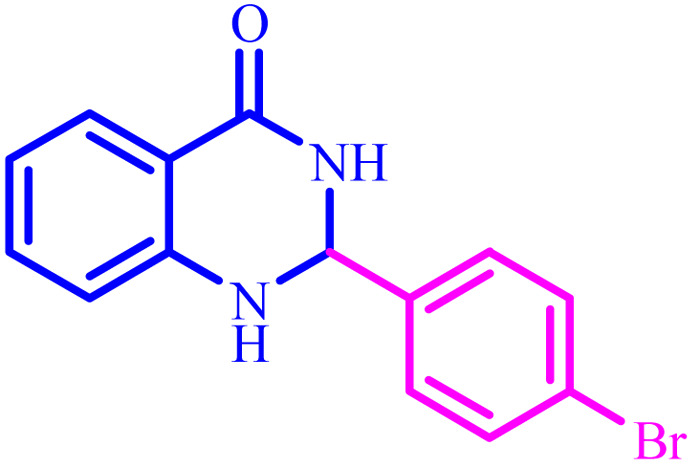	45	96
4	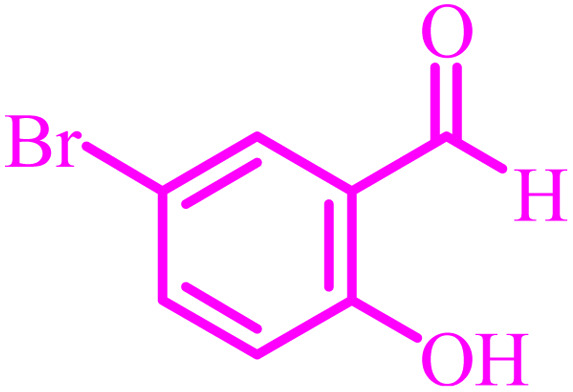	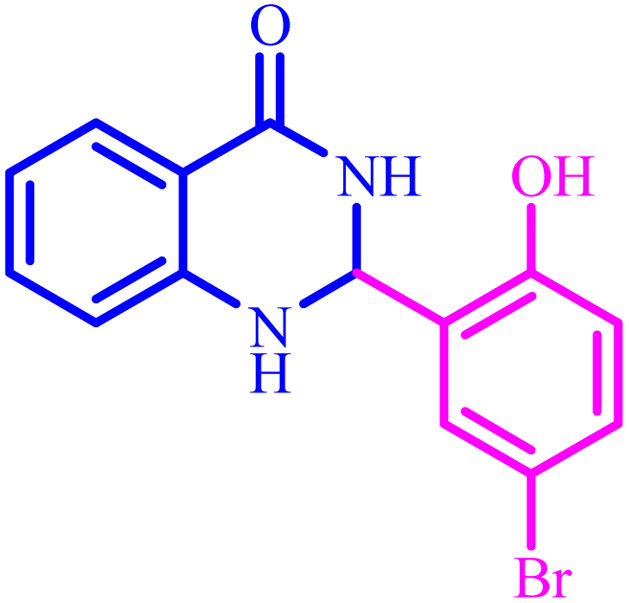	55	92
5	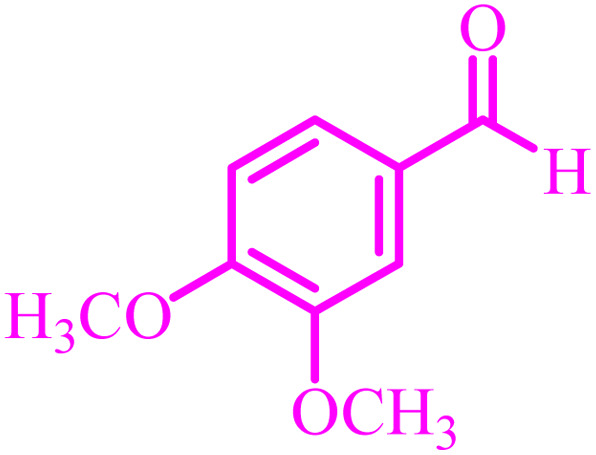	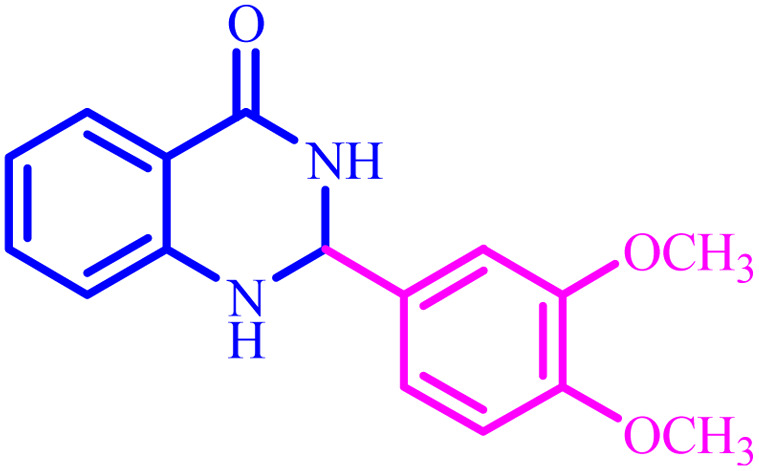	65	93
6	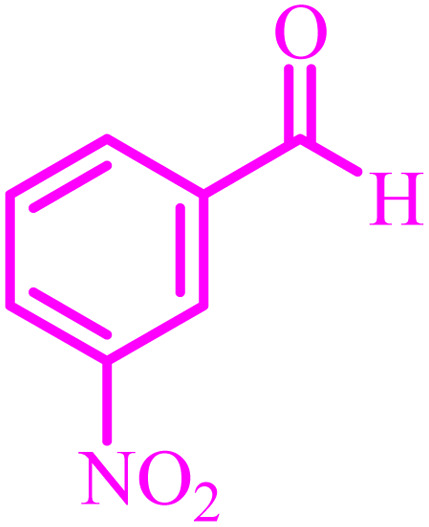	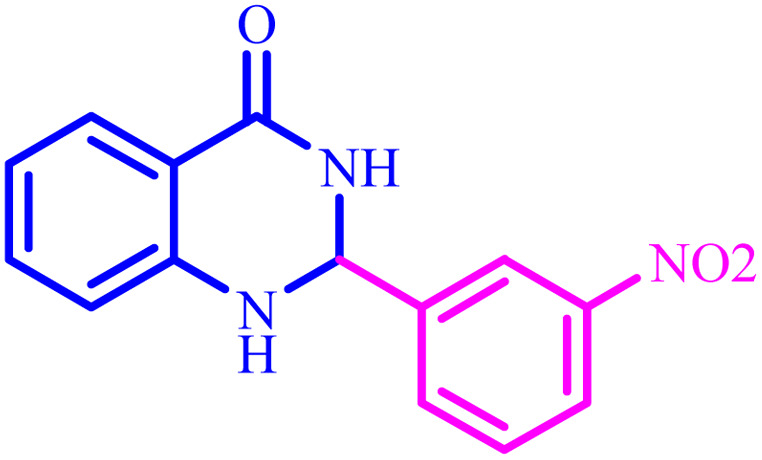	120	90
7	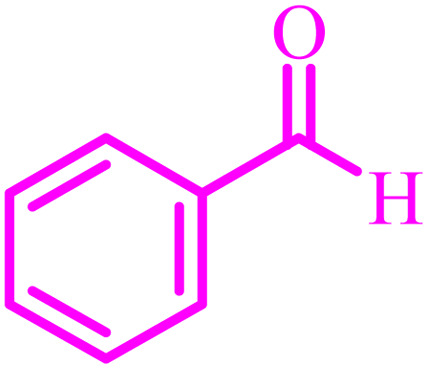	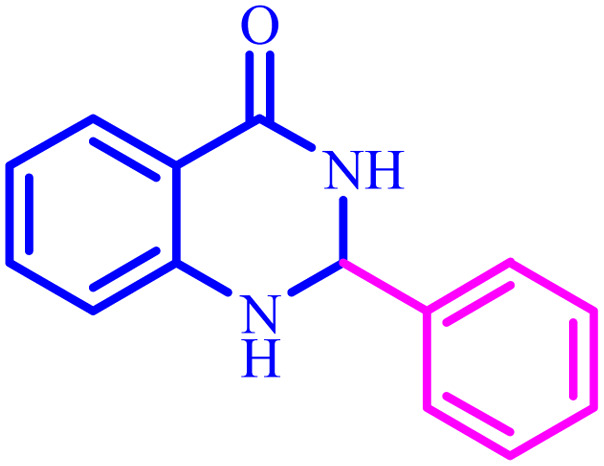	35	98
8	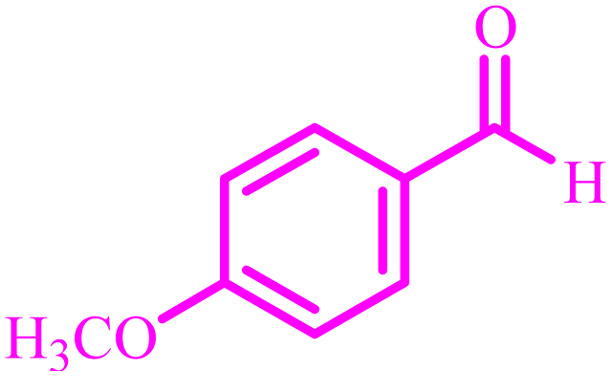	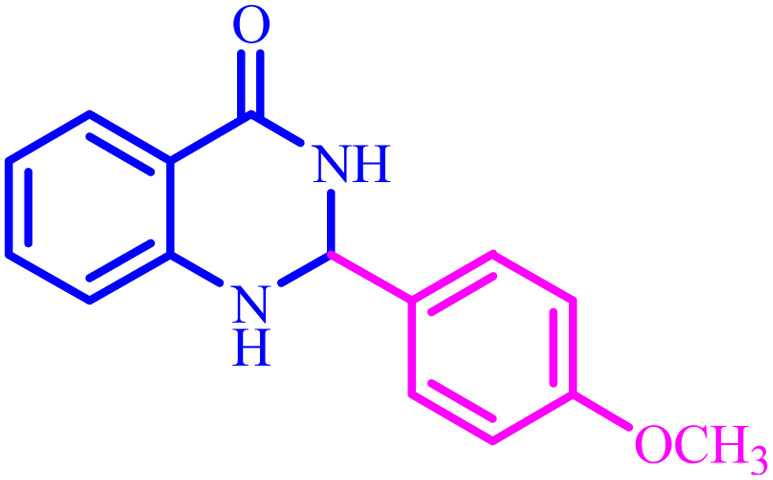	45	94
9	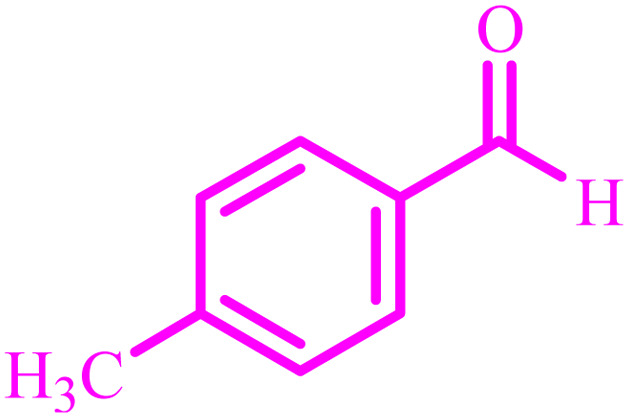	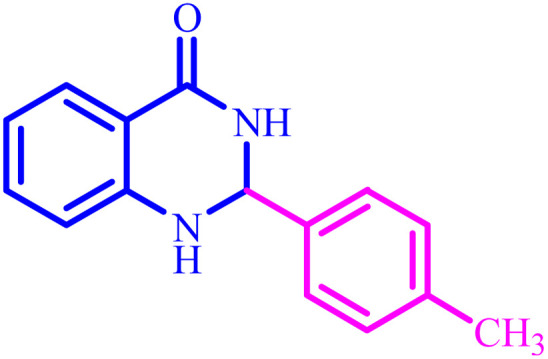	35	96
10	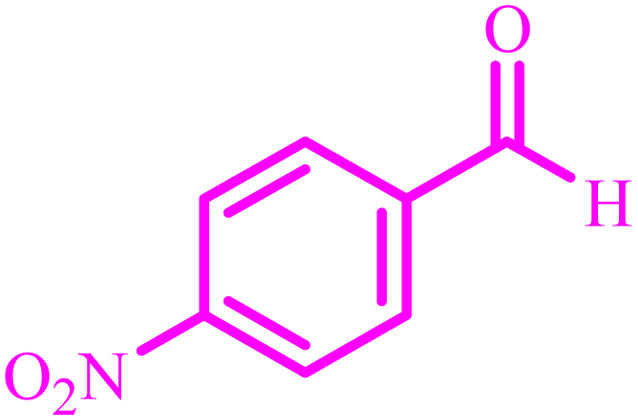	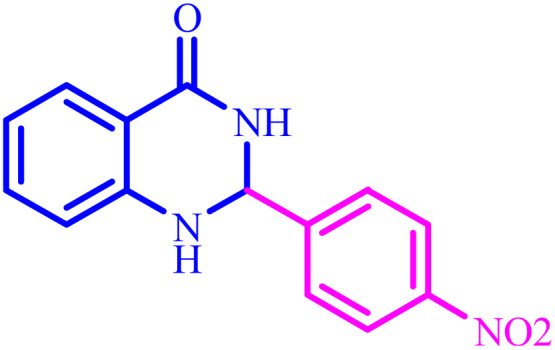	55	91
11	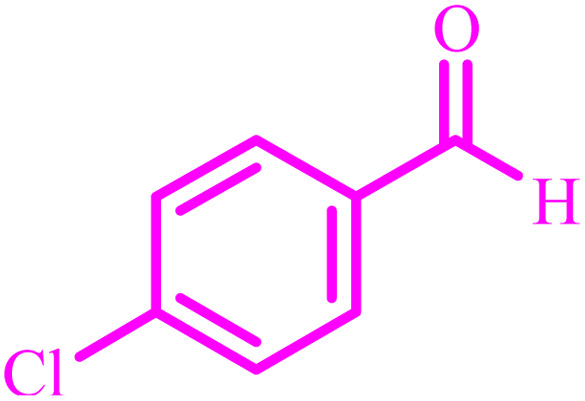	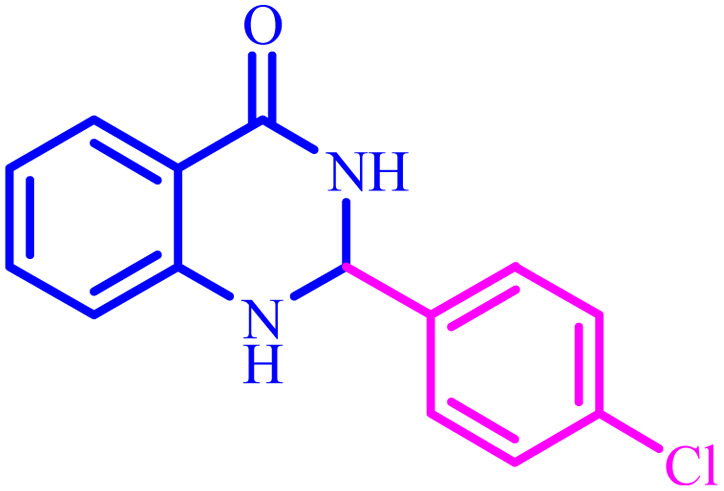	25	92

a Isolated yield.

In next attempt to find the best solvent, the same reaction in two polar protic solvents (water, ethanol, and methanol) and polar aprotic solvents (DMF, CHCl_3_, DMSO and acetonitrile) were explored (Table 3, entries 6–12), which can effectively dissolve both polar and non-polar compounds and provide a suitable environment for the reaction to occur.

As shown in Table 3, when the reaction is carried out in CHCl_3_, as a solvent, the desired product was not observed after 85 min under reflux conditions. However, when the solvents were changed to DMF, DMSO, MeOH and H_2_O, were shown moderate to good yield.

Preliminary results illustrated the maximum yields in EtOH (Table 3, entry 4). We chose EtOH as solvent for the synthesis of 2,3-dihydroquinazolin-4(1*H*)-one because it is green, safe, environmentally friendly, and easier work-up.

The optimized reaction conditions were extended to a series of aldehydes with electron donor and electron acceptor functional groups. As shown in Table 4, the corresponding 2,3-dihydroquinazolin-4(1*H*)-one derivatives were synthesized in 90–97% yields under optimized conditions.

**Scheme 2:**
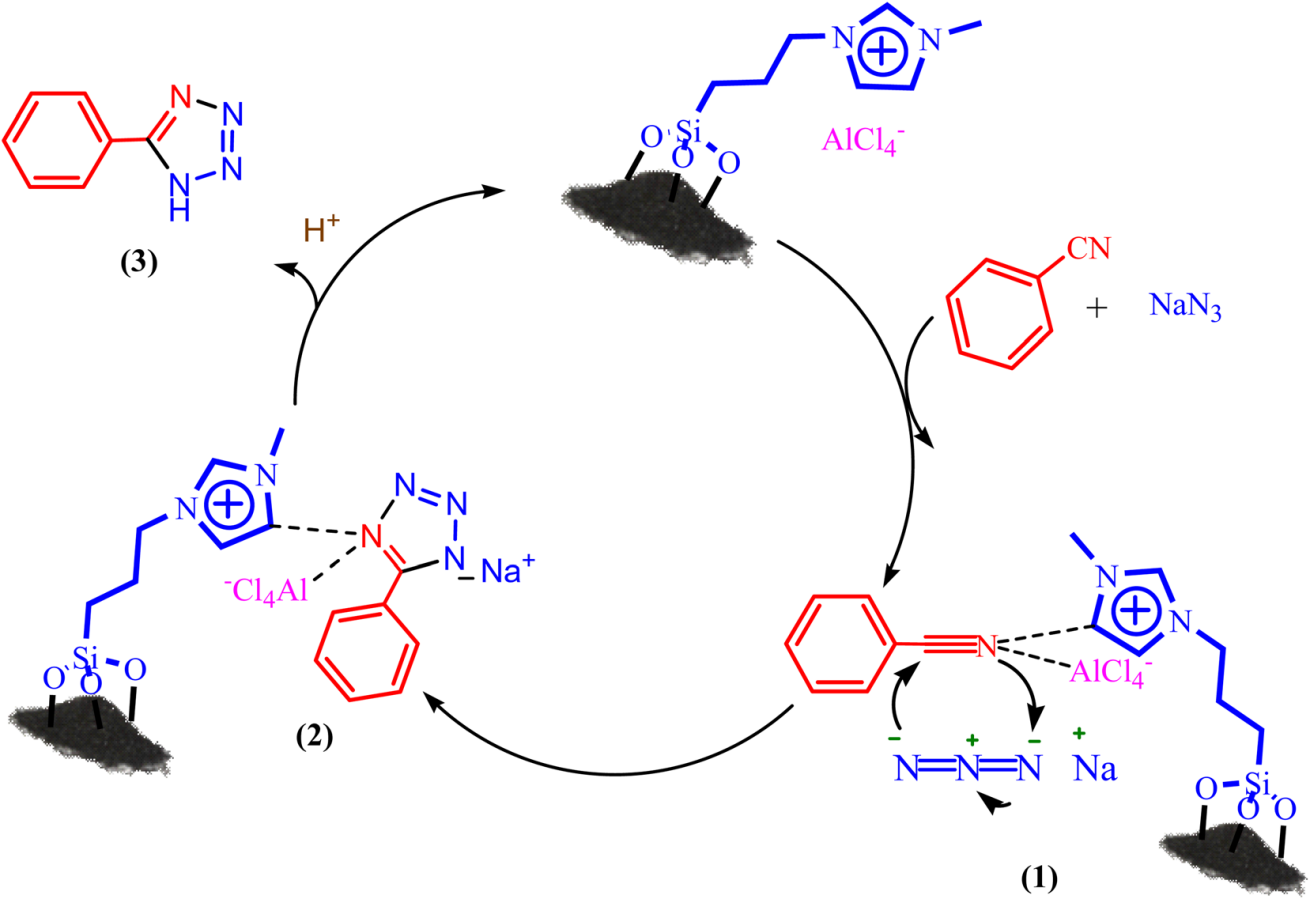
A plausible hypothetical mechanism for the formation of 5-aryl 1*H*-tetrazole catalyzed by BC/[TESPMI]AlCl_4_.

A purposed mechanism for the tetrazole formation catalyzed by BC/[TESPMI]AlCl_4_ is illustrated in Scheme 2.^56^ At first, the catalyst is coordinated with C≡N functional groups, intermediate (1) formed. N_3_ ion attacked on intermediate (1) by [3 + 2] cycloaddition reaction and formed intermediate (2), which finally formed the corresponding tetrazole (3).

**Scheme 3:**
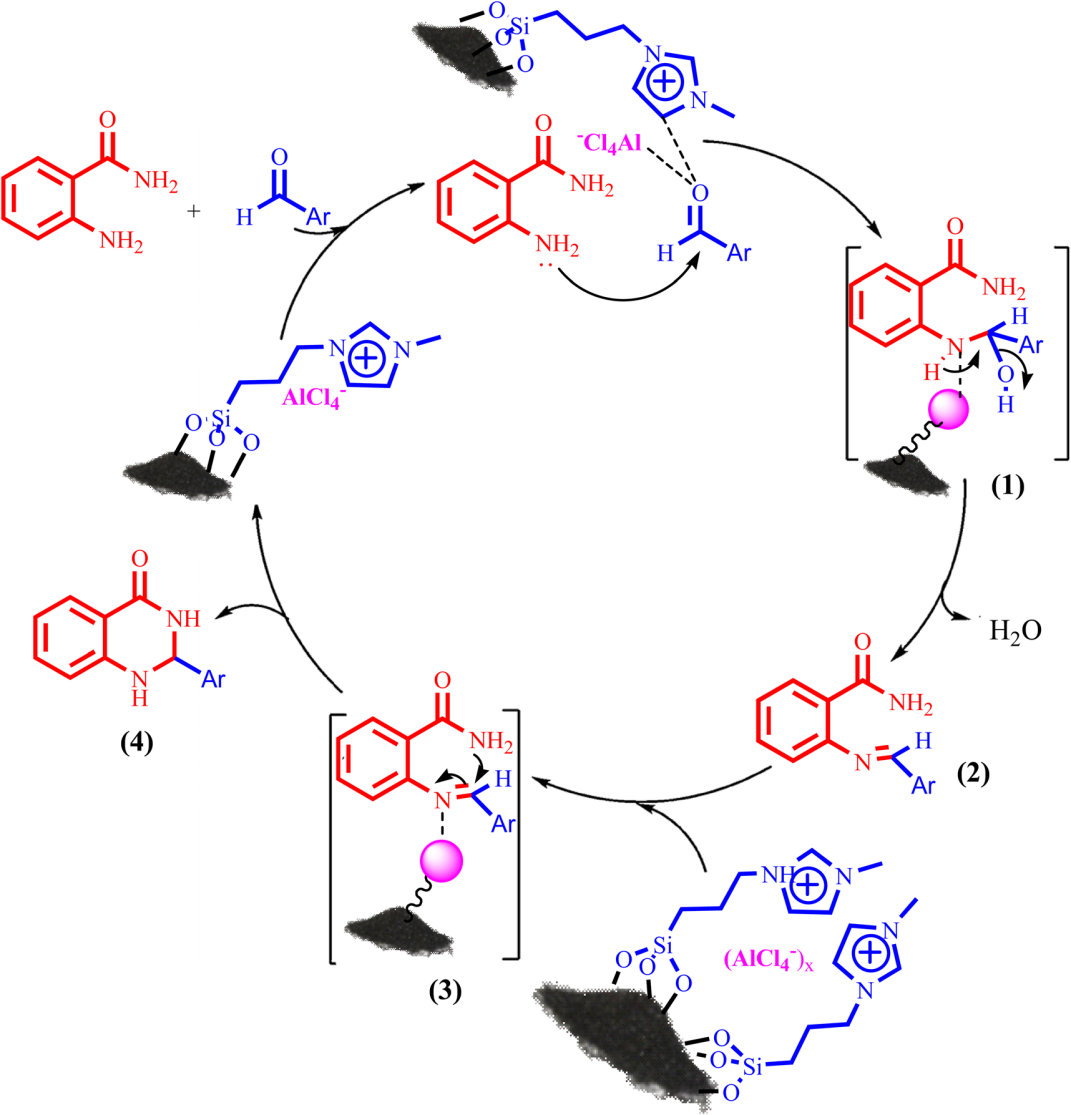
A plausible hypothetical mechanism for the formation of 2,3-dihydroquinazolin-4(1*H*)-one catalyzed by BC/[TESPMI]AlCl_4_.

Also, Scheme 3 presented a proposed mechanism pathway for the formation of 2,3-dihydroquinazolin-4(1*H*)-ones in the presence of BC/[TESPMI]AlCl_4_ catalyst. Based on this mechanism, initially, the imidazolium cations and Al-based nanocatalyst interacted with carbonyl group in aldehyde. Next, the activated carbonyl is attacked by 2-aminobenzamide's NH_2_, which imine intermediate (2) is formed. Afterwards, imine intermediate (2) and catalyst produces the intermediate (3). Finally, the target 2,3-dihydroquinazolin-4(1*H*)-ones (4) was formed by the nucleophilic cycloaddition of amide nitrogen to the imine active group.

**Fig. 9:**
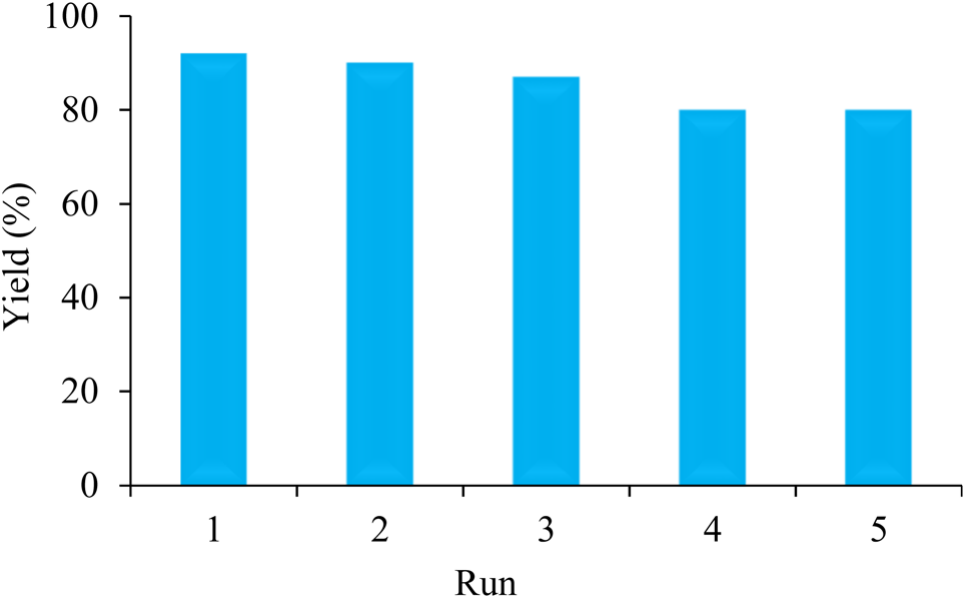
Recycling of nanocatalyst for the preparation of 2,3-dihydroquinazolin-4(1*H*)-one derivatives under optimized conditions.

The recyclability test of BC/[TESPMI]AlCl_4_ catalyst has been done to prove the heterogeneous behavior and no metal leaching of the BC/[TESPMI]AlCl_4_ catalyst in the preparation of 2,3-dihydroquinazolin 4(1*H*)-one derivatives. In this stage, the reaction between 4-chlorobenzaldehyde with 2-aminobenzamide has been tested under the optimal conditions. At the end of the reaction, the catalyst was recovered from the reaction by filtration. The recovered catalyst washed with ethanol and drying it at 50 °C. The recovered catalyst was reused in the next similar reaction. We continued this experiment up to 5 times. Finally, it was observed that the catalytic activity of BC/[TESPMI]AlCl_4_ was not reduced significantly (Fig. 9).

To assess the stability of BC/[TESPMI]AlCl_4_, the leaching of active species in the reaction mixture was examined using ICP analysis after five cycles. The findings indicated that the leaching amounts of aluminum is 0.032 mmol g^−1^, which indicates that a small accumulation may lead to a slight decline in recycled catalyst's catalytic performance. It is believed that strong interactions between the Al and the atoms of the BC/[TESPMI] play a significant role in preventing metal leaching during the reaction. This illustrates that no substantial leaching occurred during the reaction.

**Fig. 10:**
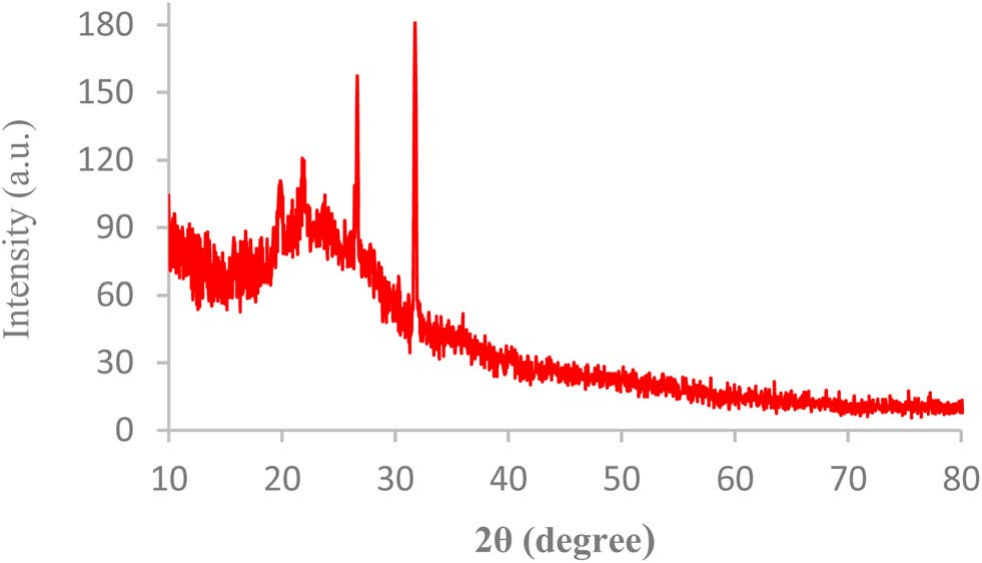
XRD pattern of BC/[TESPMI]AlCl_4_ after five times reuse.

**Fig. 11:**
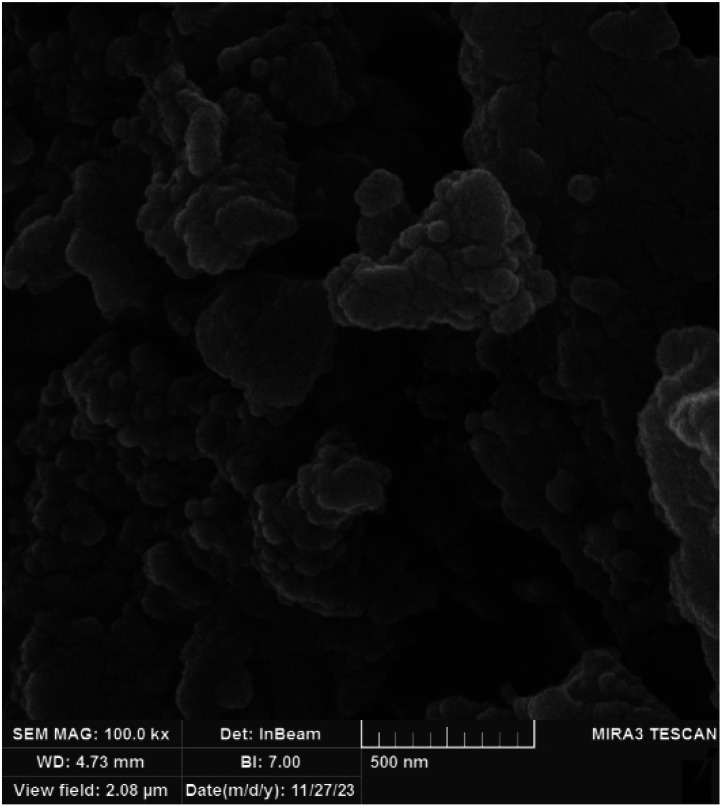
FE-SEM image of BC/[TESPMI]AlCl_4_ after five times reuse.

SEM and XRD analyses for BC/[TESPMI]AlCl_4_ were investigated after five runs. The result of XRD analysis of the reused catalyst showed that the structure of the catalyst was preserved during the reactions (Fig. 10). Fig. 11 illustrates SEM analysis of BC/[TESPMI]AlCl_4_ after five runs. As can be seen, almost the BC/[TESPMI]AlCl_4_ particles with the same size and morphology as the fresh catalyst confirm the spherical shape.

To demonstrate the benefit of BC/[TESPMI]AlCl_4_ catalyst compared to other catalysts that have been used in this field, the obtained results in this work was compared with previous catalysts. For this aim, the cycloaddition of benzonitrile and sodium azide (Table 5) and condensation of benzaldehyde with 2-aminobenzamide (Table 6) were considered. Based on the obtained data, BC/[TESPMI]AlCl_4_ catalyst can be compared with some reported catalysts in terms of efficiency and reaction time. As shown, BC/[TESPMI]AlCl_4_ catalyst provides better performance in reaction time than others. Also, BC/[TESPMI]AlCl_4_ can easily be recycled and reused.

**Table 5:** Comparison results for BC/[TESPMI]AlCl_4_ in the synthesis of 5-phenyl-1*H*-tetrazole with previously reported procedures

Entry	Catalyst	Time (min)	Yield (%)	Ref.
1	CoY zeolite	14 h	95	57
2	[Fe_3_O_4_@TAM-Schiff-base-Cu(ii)] complex	100	98	58
3	ZrO-SB-APT@MCM-41	130	89	59
4	PdArg@boehmite	420	97	60
5	Fe_3_O_4_/ZnS HNSs	24 h	86	61
6	Co-(PYT)_2_@BNPs	120	98	62
7	CuFe_2_O_4_	720	90	63
8	Cu-TBA@biochar	420	98	54
9	La-Schiff base@MCM-41	120	98	64
10	BC/[TESPMI]AlCl_4_	100	96	This work

**Table 6:** Comparison results for BC/[TESPMI]AlCl_4_ in the synthesis of 2-phenyl-2,3-dihydroquinazolin-4(1*H*)-one with previously reported procedures

Entry	Catalyst	Time (min)	Yield (%)	Ref.
1	Fe_3_O_4_–Schiff base of Cu(ii)	60	99	65
2	CuCl/Fe_3_O_4_-TEDETA	30	97	66
3	SBA/AuNP	90	91	67
4	Amberlyst-15	60	85	68
5	Cu(NO_3_)_2_/F_3_O_4_-DETA	50	97	69
6	H_3_BO_3_-MCM-41	45	90	70
7	Ni-CP	100	96	71
8	BC/[TESPMI] AlCl_4_	35	98	This work

In some reagents reported, toxic or expensive solvents are used. But in this work, PEG-400 is used as a green and environmentally friendly solvent.

## 4. Conclusions

In summary, aluminium-based ionic liquid immobilized biochar surface was used for the sustainable and green synthesis of 5-substituted 1*H*-tetrazole and 2,3-dihydroquinazolin-4(1*H*)-one derivatives as a reusable, highly effective, and eco-friendly nanocatalyst. The nanocatalyst was prepared through a three-step procedure and characterized using various techniques including EDX, BET, FT-IR, SEM, PXRD, TEM, ICP and TGA. The nanocatalyst could be easily separated from the reaction mixtures and reused up to five times while maintaining its effectiveness. This approach has several benefits, including the use of a reusable and eco-friendly catalyst, a green solvent, mild reaction conditions, low catalyst loading, and the ability to achieve high yields without requiring a chromatographic column to isolate products.

## Conflicts of interest

There is no conflict of interest for authors.

## Supplementary Material

RA-013-D3RA06440A-s001
