# Supplementary material for: Aluminium-based ionic liquid grafted on biochar as a heterogeneous catalyst for the selective synthesis of tetrazole and 2,3-dihydroquinazolin 4(1H)-one derivatives
Source: RSC Adv. 2023 Dec 6;13(50):35569–82. doi: 10.1039/d3ra06440a (PMC10699257; doi:10.1039/d3ra06440a)
Supplement: RA-013-D3RA06440A-s001 [file RA-013-D3RA06440A-s001.pdf]

Electronic Supplementary Material (ESI) for RSC Advances.
This journal is © The Royal Society of Chemistry 2023

Aluminium-based ionic liquid grafted on biochar as a heterogeneous catalyst for the selective synthesis of tetrazole and 2,3-dihydroquinazolin 4(1H)-one derivatives

Masoomeh Norouzi*[a], Parisa Moradi[a], Maryam Khanmoradi[a]

[a]*Department of Chemistry, Faculty of Science, Ilam University, P. O. Box69315516, Ilam, Iran.*

* *E-mail address:* m.norozi@ilam.ac.ir

Data of compounds

Figure S1. ^{1}HNMR spectrum of 5-(4-Chloro-phenyl)-1H-tetrazole

Figure S2. ^{1}HNMR spectrum of 5-Phenyltetrazole

Figure S3. ^{1}HNMR spectrum of 5-(3-Nitrophenyl) tetrazole

Figure S4. ^{1}HNMR spectrum of 2-(4-Bromo-phenyl)-2,3-dihydro-1H-quinazolin-4-one

Figure S5. ^{1}HNMR spectrum of 2-(5-Bromo-2-hydroxy-phenyl)-2,3-dihydro-1H-quinazolin-4-one

Figure S6. ^{1}HNMR spectrum of 2-(3,4-Dimethoxy-phenyl)-2,3-dihydro-1H-quinazolin-4-one

Figure S7. ^{1}HNMR spectrum of 2-Phenyl-2,3-dihydro-1H-quinazolin-4-one

Figure S8. ^{1}HNMR spectrum of 2-(4-Chloro-phenyl)-2,3-dihydro-1H-quinazolin-4-one

5-(4-Chloro-phenyl)-1H-tetrazole

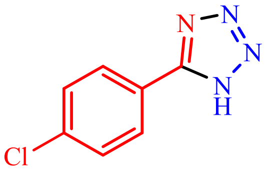

^{1}H NMR (400 MHz, DMSO) δ: 16.97 (s, 1H, NH), 8.06-8.03 (d, 1H, J=8 Hz, CH), 7.72-7.67 (d, 1H, J=8 Hz, Ar-H).

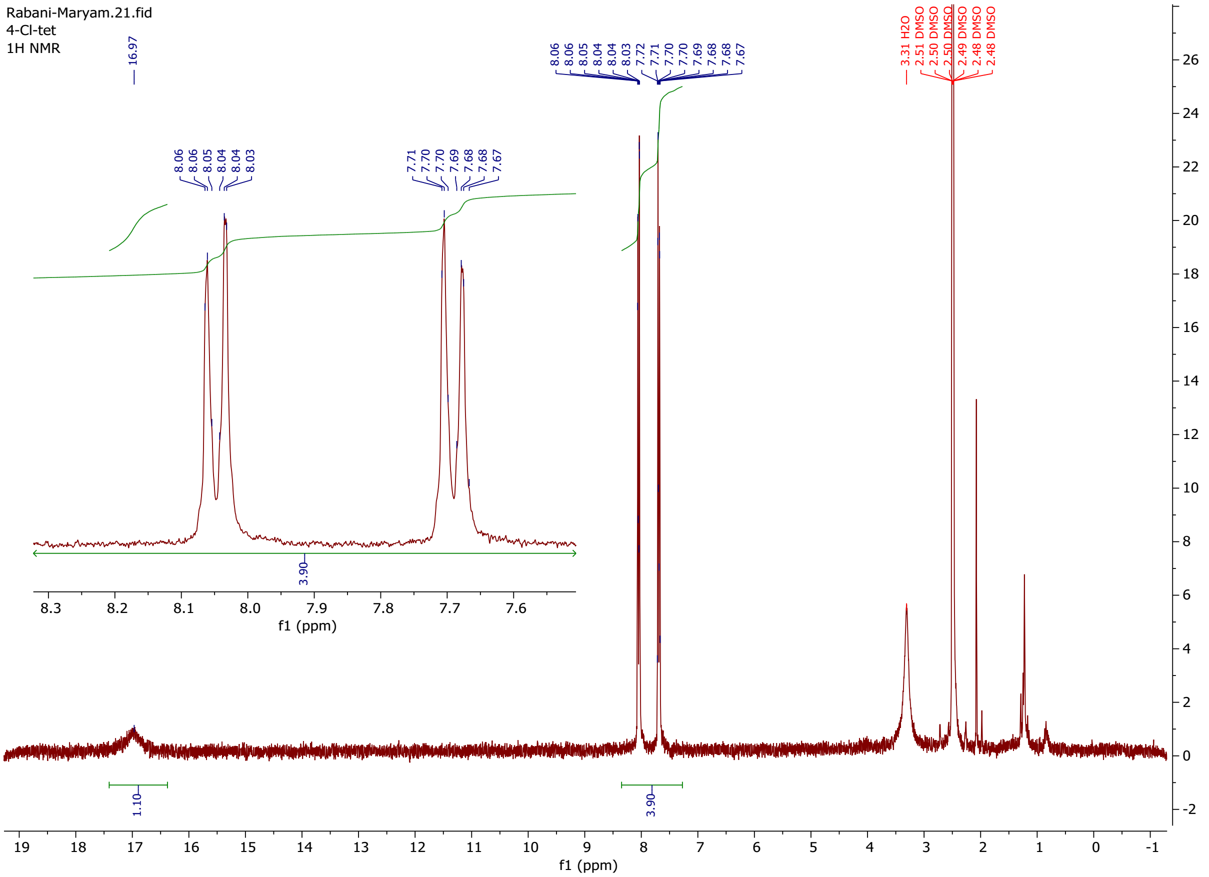

Figure S1. ^{1}HNMR spectrum of 1HNMR spectrum of 5-(4-Chloro-phenyl)-1H-tetrazole

5-Phenyltetrazole

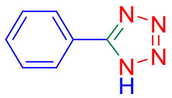

^{1}H NMR (400 MHz, DMSO) δ: 8.10-8.05 (t, J = 8 Hz, 2H), 7.94-7.86 (t, J = 8 Hz, 1H), 7.80-7.74 (t, J = 8 Hz, 2H).

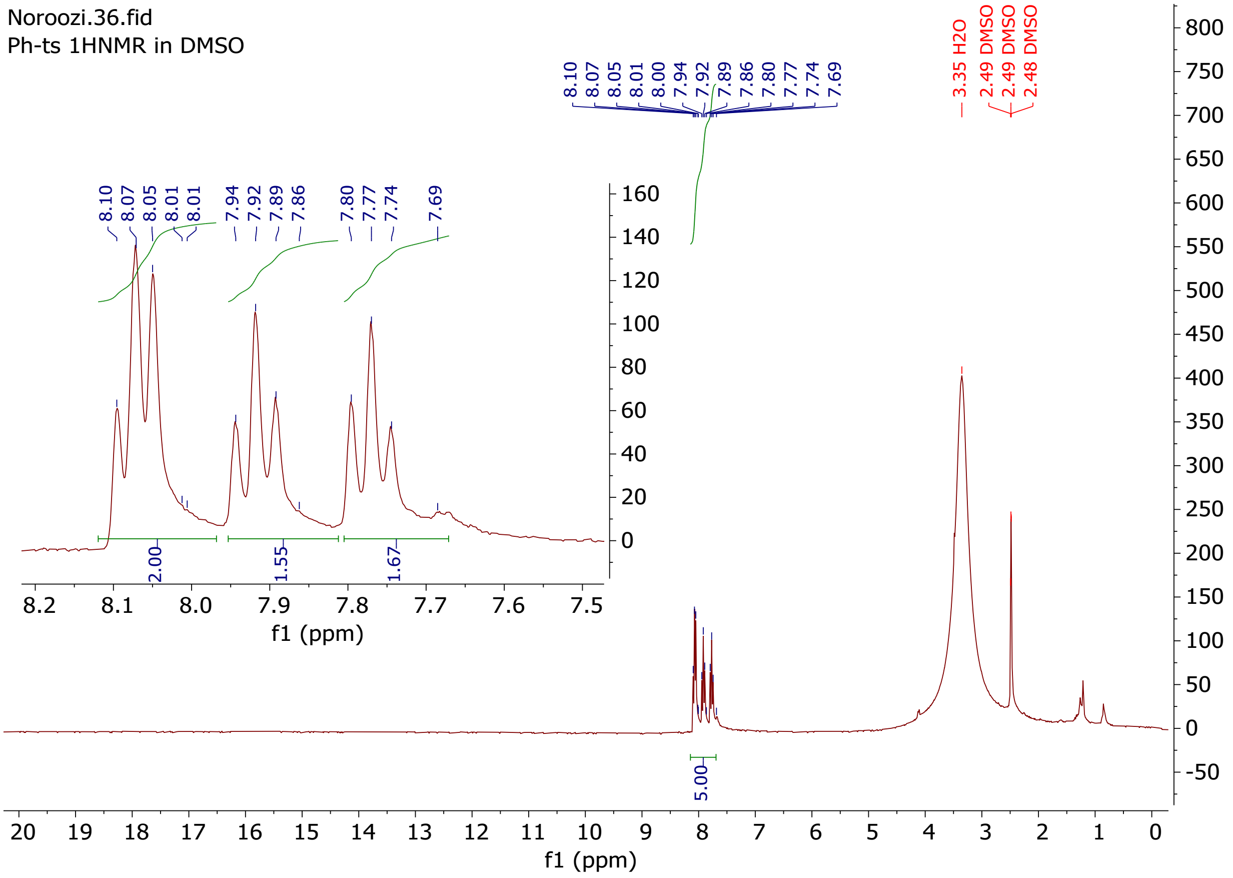

Figure S2. ^{1}HNMR spectrum of 5-Phenyltetrazole

5-(3-Nitrophenyl)tetrazole

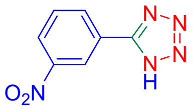

^{1}H NMR (400 MHz, DMSO) δ: 8.82 (s, 1H), 8.54 – 8.34 (m, 2H), 7.92-7.86 (t, J = 8. Hz, 1H).

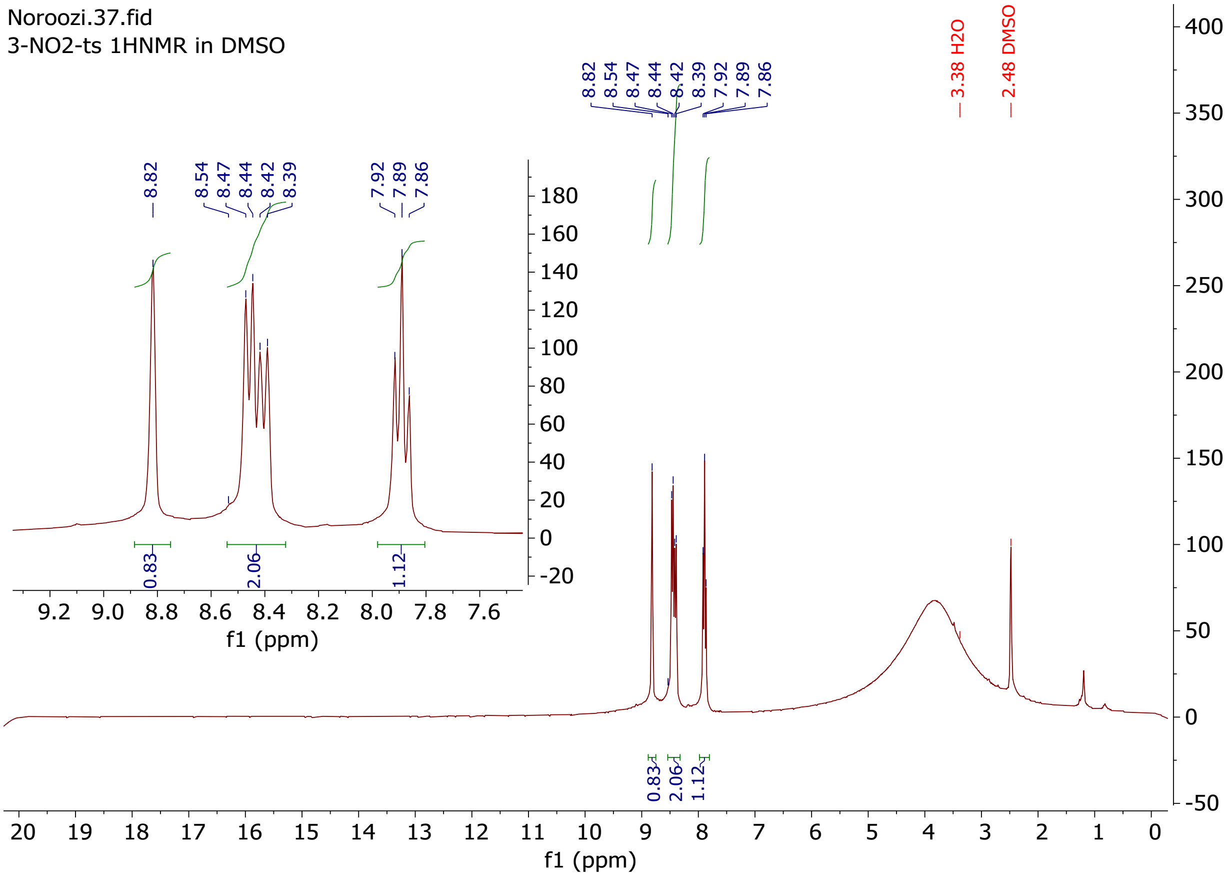

Figure S3. ^{1}HNMR spectrum of 5-(3-Nitrophenyl)tetrazole

2-(4-Bromo-phenyl)-2,3-dihydro-1H-quinazolin-4-one

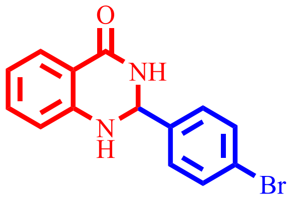

^{1}H NMR (400 MHz, DMSO-d_6): δ 8.31 (s, 1H), 7.60-7.56 (d, J= 7.6 Hz, 3H), 7.44-7.41 (d, J = 7.4 Hz, 2H), 7.26-7.21 (s, J=7 Hz, 1H), 7.13 (s, 1H), 6.75-6.4 (m, 2H), 5.74 (s, 1H) ppm.

.

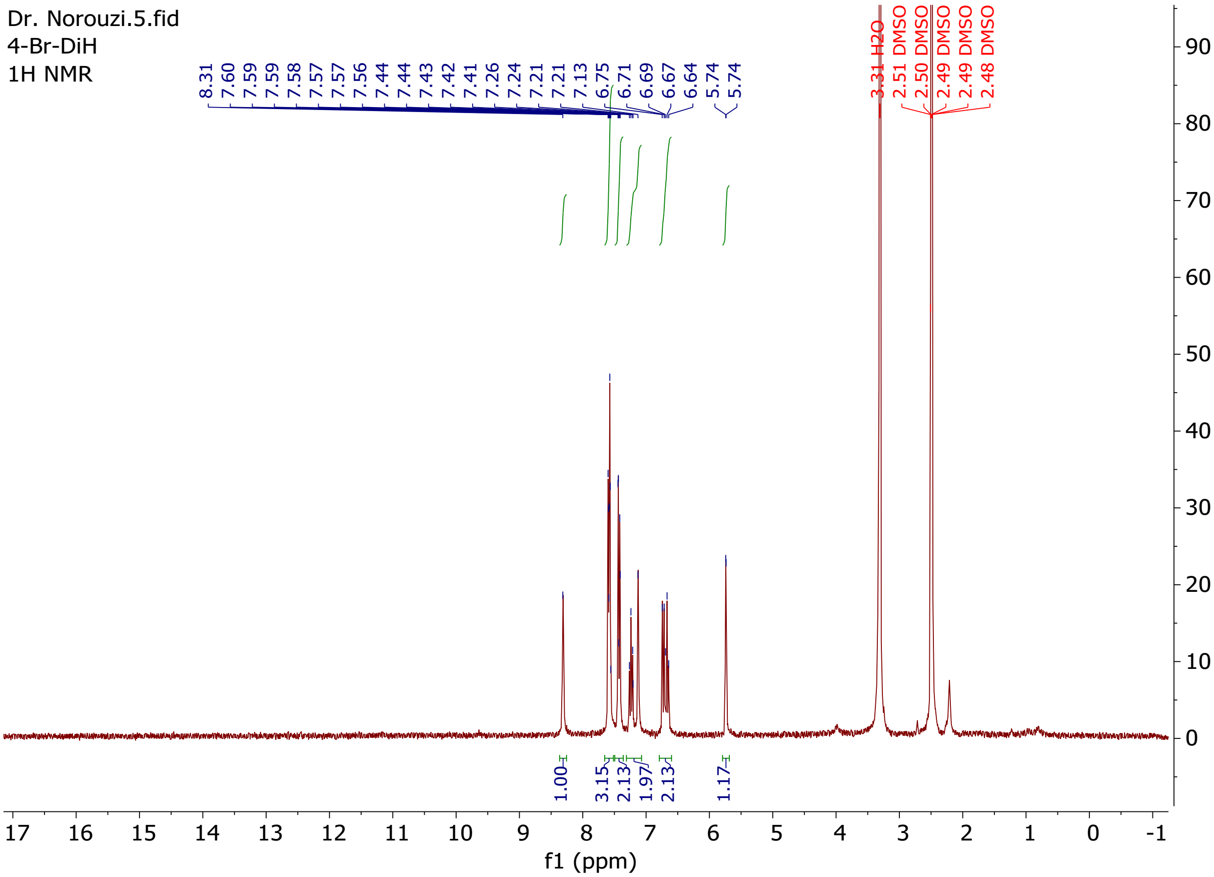

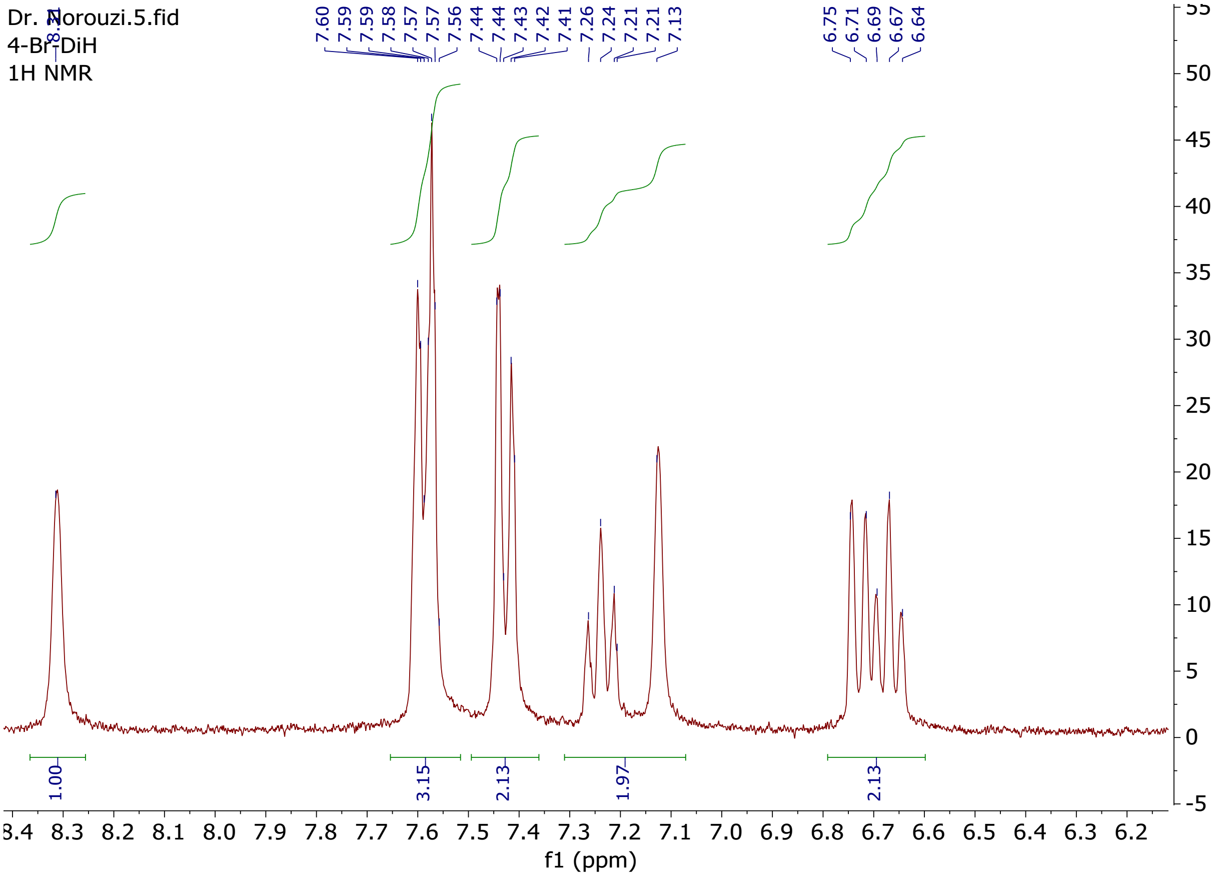

Figure S4. ^{1}HNMR spectrum of 2-(4-Bromo-phenyl)-2,3-dihydro-1H-quinazolin-4-one

2-(5-Bromo-2-hydroxy-phenyl)-2,3-dihydro-1H-quinazolin-4-one

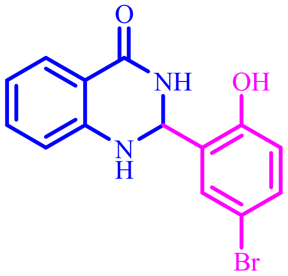

^{1}H NMR (400 MHz, DMSO-d_6): δ 10.22 (s, 1H), 7.99 (s, 1H), 7.63-7.60 (d, *J*= 7.6 Hz, 1H), 7.30-7.20 (m, 2H), 6.83-6.65 (m, 4H), 5.95 (s, 1H) ppm.

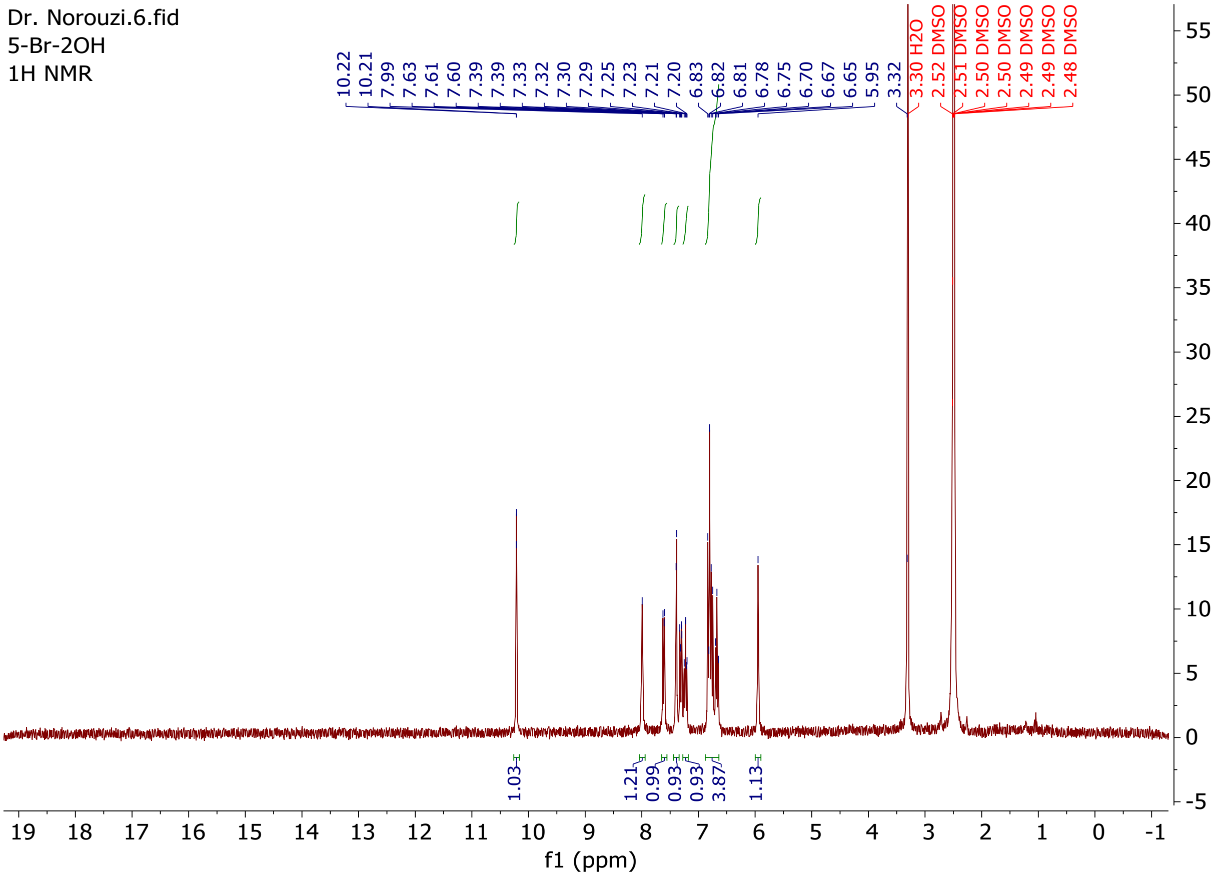

Figure S5. ^{1}HNMR spectrum of 2-(5-Bromo-2-hydroxy-phenyl)-2,3-dihydro-1H-quinazolin-4-one

2-(3,4-Dimethoxy-phenyl)-2,3-dihydro-1H-quinazolin-4-one

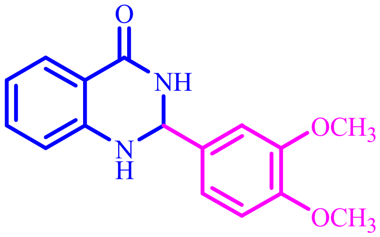

^{1}H NMR (400 MHz, DMSO-*d6*): δ 8.12 (s, 1H), 7.62-7.59 (d, *J*= 7.6, 1H), 7.26-7.21 (t, *J*= 7.2, 1H), 7.20 (s, 1H), 7.01-6.92 (m, 3H), 6.73-6.76 (d, *J*= 6.7 Hz, 1H), 6.96-6.64 (t, *J*=6.7 Hz, 1H), 5.68 (s, 1H), 3.75 (s, 6H) ppm.

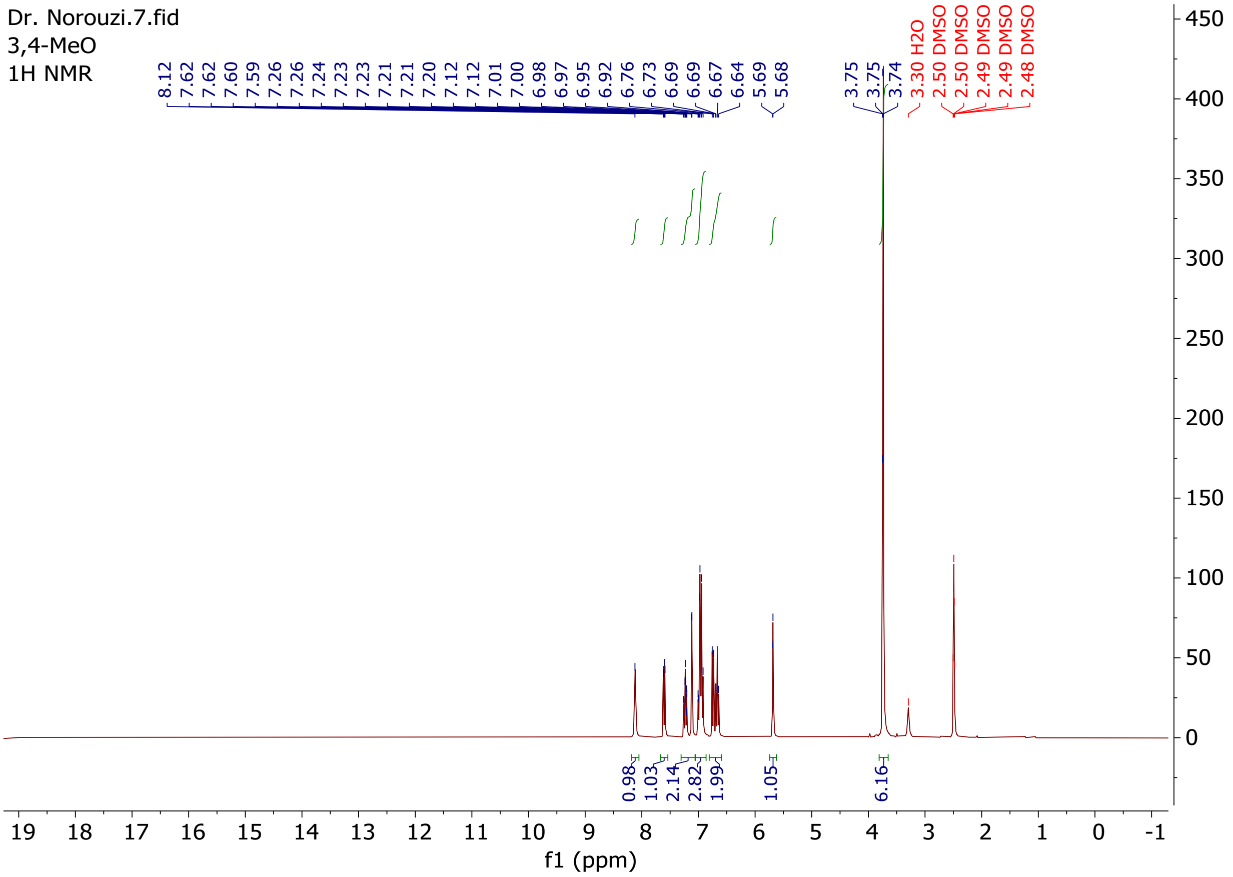

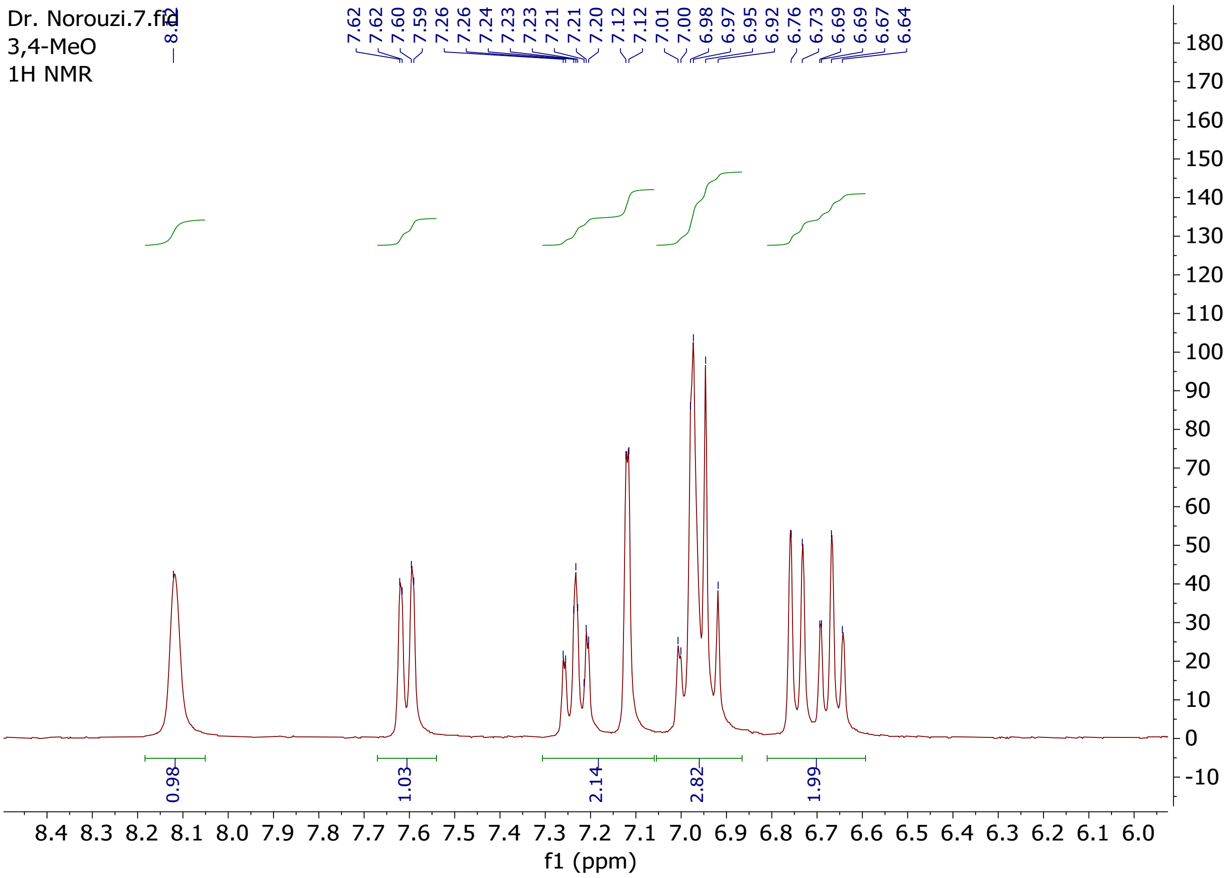

Figure S6. ^{1}HNMR spectrum of 2-(3,4-Dimethoxy-phenyl)-2,3-dihydro-1H-quinazolin-4-one

2-Phenyl-2,3-dihydro-1H-quinazolin-4-one

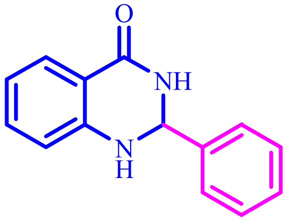

^{1}H NMR (400 MHz, DMSO): δ 8.25 (s, 1H), 7.61-7.57 (d, J = 7.6 Hz, 1H), 7.49-7.46 (d, J = 7.6 Hz, 2H), 7.41-7.34 (m, 3H), 7.28-7.30 (t, J = 7.0 Hz, 1H), 7.08 (s, 1H), 6.74-6.70 (d, J = 7.0 Hz, 1H), 6.69-6.64 (t, J= 7.0 Hz, 1H), 5.74 (s, 1H).

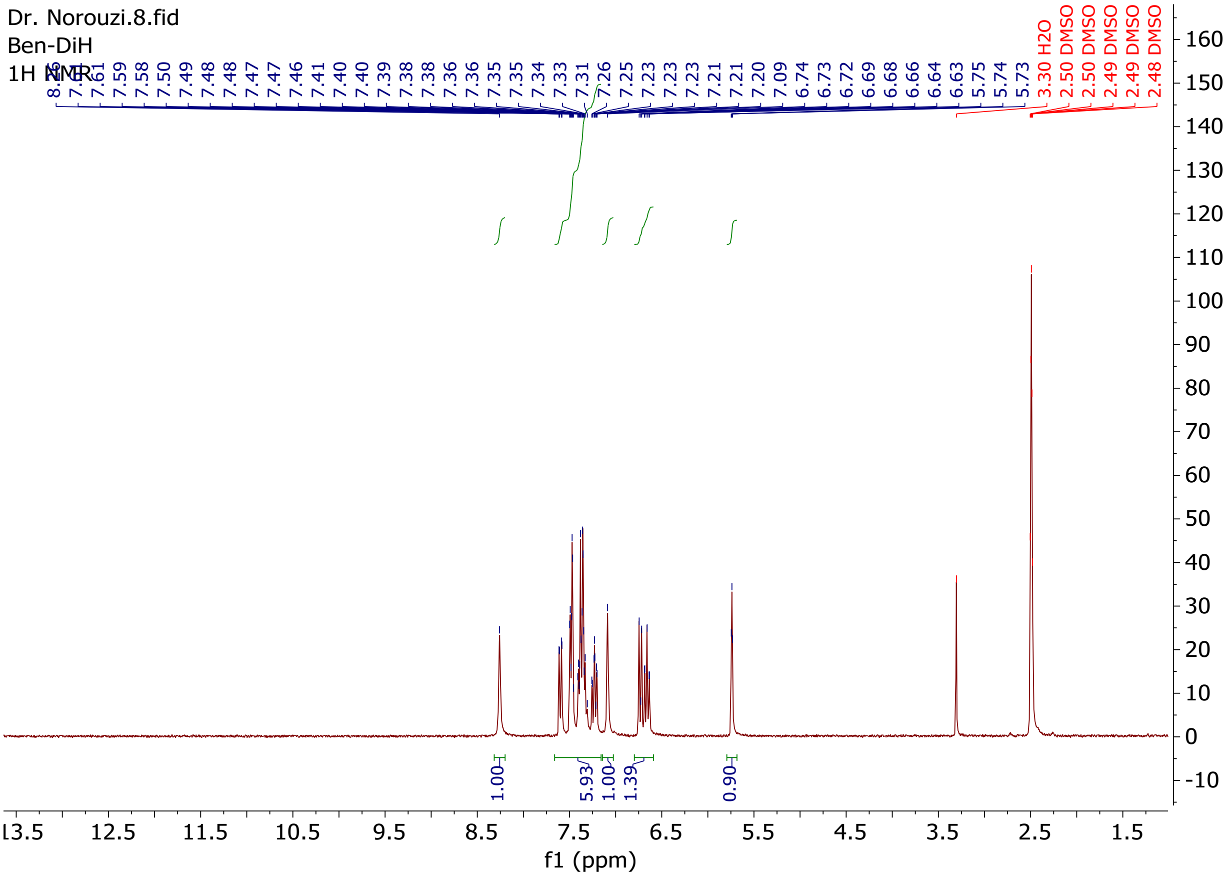

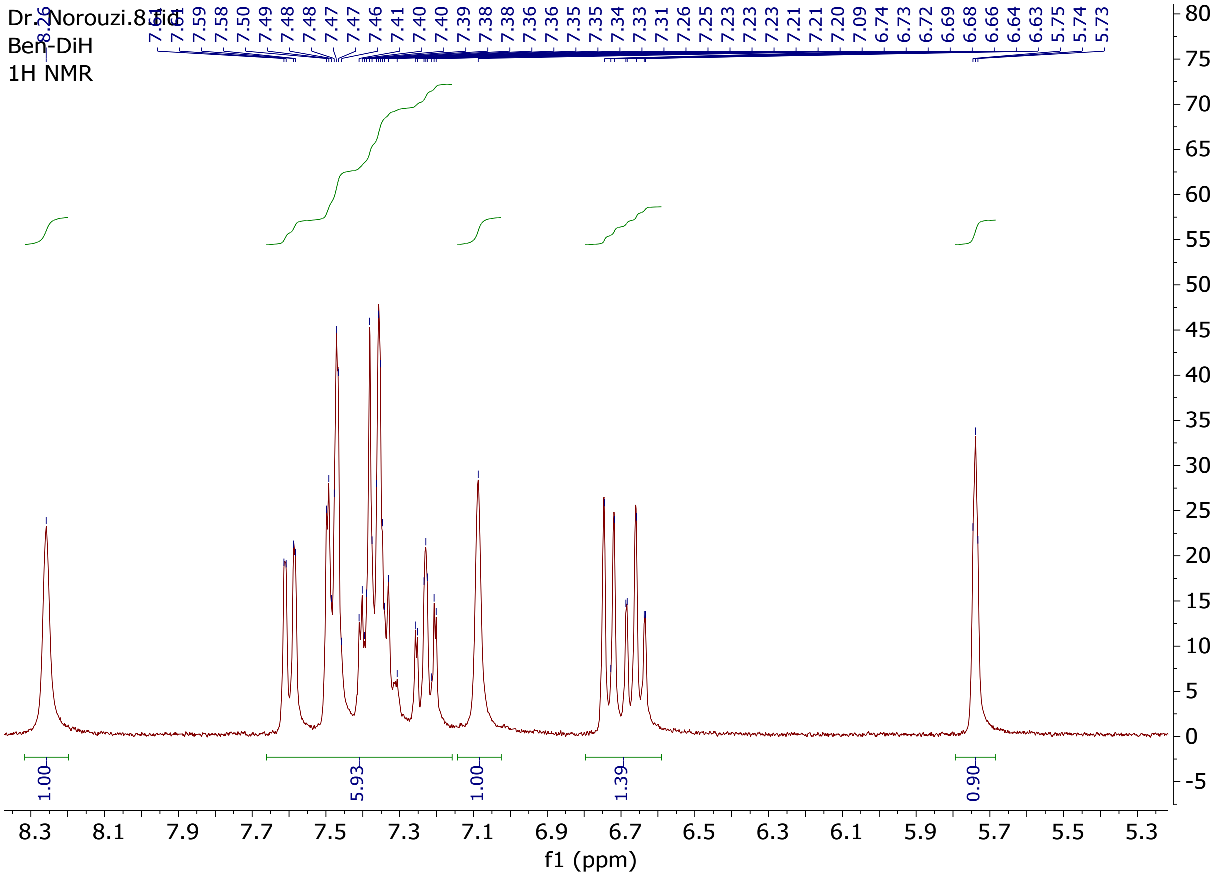

Figure S7. ^{1}HNMR spectrum of 2-Phenyl-2,3-dihydro-1H-quinazolin-4-one

2-(4-Chloro-phenyl)-2,3-dihydro-1H-quinazolin-4-one

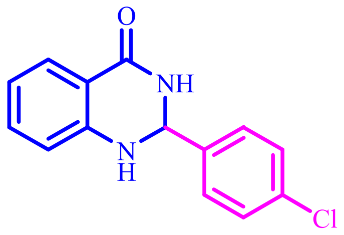

^{1}H NMR (400 MHz, DMSO-d_6): δ 8.31 (s, 1H), 7.62-7.55 (d, J = 7.1 Hz, 1H), 7.52-7.40 (m, 4H), 7.26-7.20 (t, 1H), 7.12 (s, 1H), 6.75-6.70 (d, J=7.0, 1H), 6.71-6.64 (t, J=7.0, 1H), 5.75 (s, 1H) ppm.

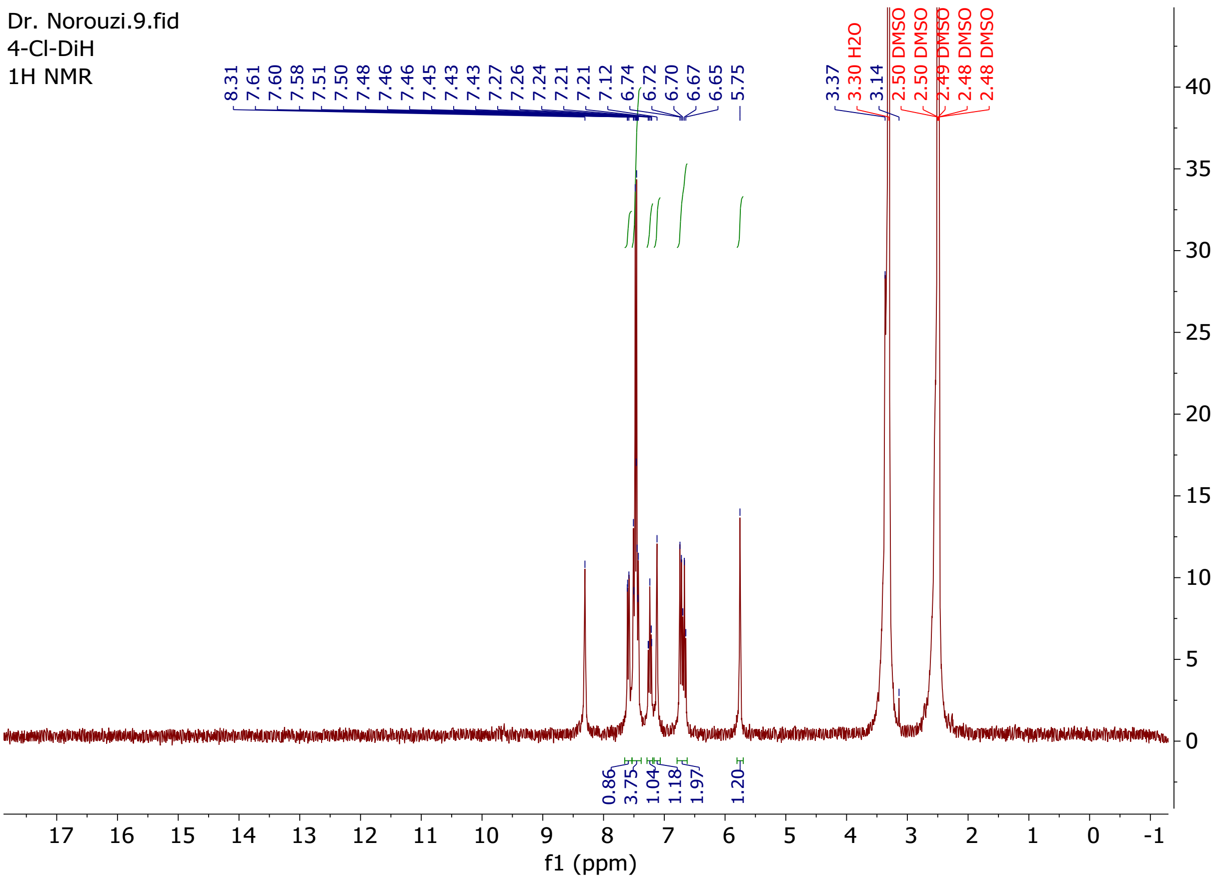

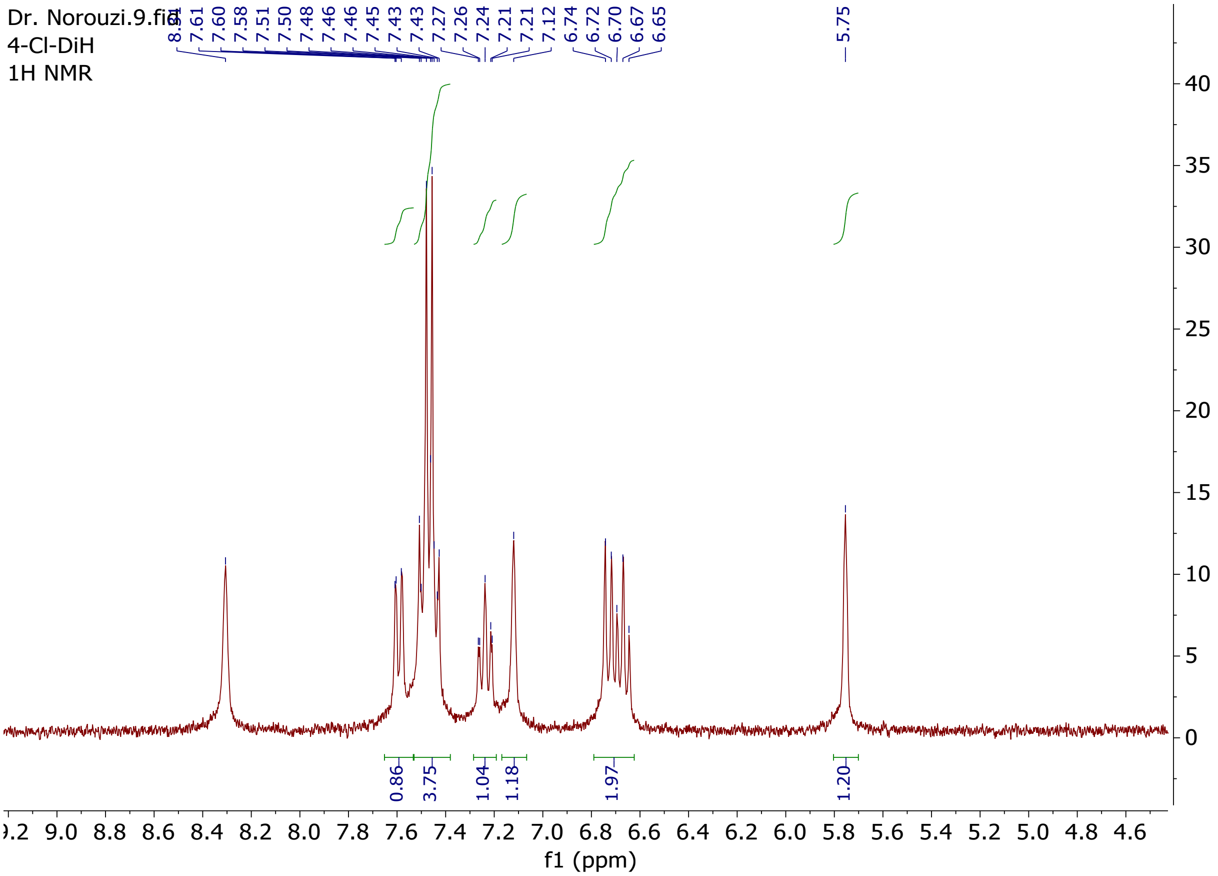

Figure S8. ^{1}HNMR spectrum of 2-(4-Chloro-phenyl)-2,3-dihydro-1H-quinazolin-4-one
